# hnRNPK-regulated *LINC00263* promotes malignant phenotypes through miR-147a/CAPN2

**DOI:** 10.1038/s41419-021-03575-1

**Published:** 2021-03-17

**Authors:** Woo Joo Lee, Chang Hoon Shin, Haein Ji, Seong Dong Jeong, Mi-So Park, Hong-Hee Won, Poonam R. Pandey, Dimitrios Tsitsipatis, Myriam Gorospe, Hyeon Ho Kim

**Affiliations:** 1 Department of Health Sciences and Technology, Samsung Advanced Institute for Health Sciences and Technology, Sungkyunkwan University, Seoul, 06351 Republic of Korea; 2 Department of Digital Health, Samsung Advanced Institute for Health Sciences and Technology, Sungkyunkwan University, Seoul, 06351 Republic of Korea; 3 Laboratory of Genetics and Genomics, National Institute on Aging Intramural Research Program, National Institutes of Health, Baltimore, MD 21224 USA; 4 Research Institute for Future Medicine, Samsung Medical Center, Seoul, 06351 Republic of Korea

**Keywords:** Metastasis, Oncogenes

## Abstract

Malignant characteristics of cancers, represented by rapid cell proliferation and high metastatic potential, are a major cause of high cancer-related mortality. As a multifunctional RNA-binding protein, heterogeneous nuclear ribonucleoprotein K (hnRNPK) is closely associated with cancer progression in various types of cancers. In this study, we sought to identify hnRNPK-regulated long intergenic non-coding RNAs (lincRNAs) that play a critical role in the regulation of cancer malignancy. We found that hnRNPK controlled malignant phenotypes including invasiveness, proliferation, and clonogenicity. RNA sequencing and functional studies revealed that *LINC00263*, a novel target of hnRNPK, is involved in the oncogenic functions of hnRNPK. Knockdown of *LINC00263* mitigated the malignant capabilities. Conversely, increased malignant phenotypes were observed in *LINC00263*-overexpressing cells. Since *LINC00263* was mainly localized in the cytosol and highly enriched in Argonaute 2-immunoprecipitation (Ago2-IP), we hypothesized that *LINC00263* acts as a competitive endogenous RNA (ceRNA), and thus sought to identify *LINC00263*-associated microRNAs. Using small RNA sequencing followed by antisense oligonucleotide pull-down, miR-147a was selected for further study. We found that miR-147a negatively regulates *LINC00263* via direct interaction, thus suppressing malignant capabilities. Moreover, knockdown of hnRNPK and *LINC00263* upregulated miR-147a, indicating that *LINC00263* serves as a ceRNA for miR-147a. By analyzing RNA sequencing data and miRNA target prediction, calpain 2 (*CAPN2*) was identified as a putative target of miR-147a. Ago2-IP and luciferase reporter assay revealed that miR-147a suppressed CAPN2 expression by directly binding to the 3′UTR of *CAPN2* mRNA. In addition, we found that the weakened malignant capabilities following knockdown of hnRNPK or *LINC00263* were restored by miR-147a inhibition or CAPN2 overexpression. Furthermore, our findings were validated in various other types of cancer cells including lung cancer, colorectal cancer, neuroblastoma, and melanoma. Collectively, we demonstrate that hnRNPK-regulated *LINC00263* plays an important role in cancer malignancy by acting as a miR-147a decoy and thus upregulating CAPN2.

## Introduction

Malignant properties of cancer cells, including their highly aggressive characteristics, are major obstacles in the successful treatment of cancer. In fact, the degree of malignancy is a major factor that affects cancer mortality^1^. The limitless growth of cancer cells is a result of the abnormal activation of oncogenic signals that enhance the proliferation rate and inhibit apoptotic processes^2^. Cancer metastasis involves multiple steps in which cancer cells are disseminated from the primary site to other tissues or organs far from where they first occurred. Through the control of metastasis-associated genes, primary tumor cells prepare for metastasis by acquiring invasive capacity and proliferative properties to disseminate and survive in the secondary sites^3^. Rapid proliferation and metastatic potential are the main phenotypes associated with cancer malignancy. Therefore, the control of proliferation and metastasis is considered a promising approach for the development of cancer therapeutics.

Heterogeneous nuclear ribonucleoprotein K (hnRNPK) is a DNA- and RNA-binding protein (RBP) that contains three K homology (KH) domains, a nuclear shuttling (KNS) domain, and a nuclear localization signal (NLS)^4^. HnRNPK controls the expression of target genes mainly by directly binding to the untranslated region (UTR) of the target mRNAs. Through a wide range of regulatory mechanisms, including post-transcriptional gene regulation, hnRNPK is known to induce genes involved in the extracellular matrix, cell motility, and angiogenesis^5,6^. Furthermore, a loss-of-function screening using randomized intracellular antibodies has revealed that hnRNPK is a potential target for metastasis therapy and its cytoplasmic accumulation is crucial for its role in metastasis^7^. We previously reported that hnRNPK regulates the proliferation of cancer cells by targeting polo-like kinase 1 (*PLK1*) and heme oxygenase-1 (*HO-1*). Further, we demonstrated that mechanically, hnRNPK competes for interaction with *PLK1* mRNA^8^ and increases the expression of *HO-1* through *PTOV1*-miR-1207-5p^9^.

MicroRNAs (miRNAs) typically regulate gene expression at the post-transcriptional level by recognizing miRNA-recognition elements (MREs) within their target mRNA. Noncoding RNAs (ncRNAs) may share MREs with target mRNA of coding genes and therefore be targeted by miRNAs. This interaction and sequestering of miRNA by ncRNAs constitutes the basis for the competitive endogenous RNA (ceRNA) theory^10^. Recently, long ncRNAs (lncRNAs) have received increasing attention for their key roles in cancer progression as oncogenes and tumor suppressors^11,12^. Emerging evidence suggests that the ceRNA-mediated gene regulatory network is closely associated with cancer progression in various types of cancers.

In this study, we screened hnRNPK-regulated lncRNAs that are responsible for the oncogenic function of hnRNPK. *LINC00263* was identified as a novel target of hnRNPK and potentiates malignant properties including proliferation and invasiveness by functioning as a decoy for miR-147a and thus upregulating calpain 2 (*CAPN2*) expression.

## Materials and methods

### Cell culture and transfection

Human cervical cancer (HeLa) cells were maintained in Dulbecco’s modified Eagle’s medium (Hyclone, Logan, UT, USA). Human non-small cell lung cancer (H460 and H1299), human colon cancer (DLD1 and LoVo), human melanoma (A375P), and human neuroblastoma (T98G and A172) cells were maintained with RPMI 1640 medium (GIBCO-BRL, Grand Island, NY, USA). Both culture media were supplemented with 10% fetal bovine serum (GIBCO-BRL, Grand Island, NY) and 1% antibiotic–antimycotic solution (GIBCO-BRL). All cell lines were recently authenticated by the STR profiling and regularly tested for mycoplasma contamination. For siRNA transfection, cells were plated at 60% density and transfected with the indicated siRNAs using Lipofectamine2000 (Invitrogen, Thermo Fisher Scientific, Waltham, MA) according to the manufacturer’s protocol. The siRNAs for *HNRNPK* and *LINC00263* were synthesized by Bioneer (Daejeon, Republic of Korea; sequences are shown in Supplementary Table 1). *CAPN2*-targeting siRNA was purchased from Santa Cruz Biotechnology (sc-41459: Santa Cruz, CA). Precursor miR-147a (pre-miR-147a: PM10020) and antisense miR-147a (anti-miR-147a: AM10020) were purchased from Ambion (Ambion, Thermo Fisher Scientific, Waltham, MA) and used for overexpression or inhibition of miR-147a, respectively, using Lipofectamine2000 (Invitrogen).

### Western blot analysis

Cells were lysed using a radioimmunoprecipitation (RIPA) buffer containing protease and phosphatase inhibitors (Roche, Basel, Switzerland). Equal amounts of the cell lysate were separated by sodium dodecyl sulfate-polyacrylamide gel electrophoresis and transferred to polyvinylidene difluoride membranes (Millipore, Billerica, MA). After blocking with 5% skim milk, the membranes were incubated with the indicated primary antibody (Supplementary Table 2), washed with tris-buffered saline containing tween-20, and incubated with the appropriate secondary antibody. The protein bands were detected using an enhanced chemiluminescent reagent. GAPDH was used as a loading control.

### Reverse transcription-quantitative polymerase chain reaction (RT-qPCR) analysis

Total RNA was isolated using TRIzol (Invitrogen) according to the manufacturer’s instructions and used as a template to synthesize cDNA, using the SuperScript III First-Strand Synthesis System (Invitrogen). The expression levels of mRNAs were quantified by RT-qPCR analysis with appropriate primers (sequences are shown in Supplementary Table 3) using the Power SYBR Green PCR Master Mix (Applied Biosystems, Foster City, CA). To determine the stability of *LINC00263*, cells were transfected with control and HNRNPK siRNA. Following treatment of actinomycin D (0.5 μg/ml), cells were harvested at the indicated times and the levels of *LINC00263* and *GAPDH* mRNA were determined by RT-qPCR analysis.

### Determination of malignant phenotypes

The invasive ability of the cells was determined using the BD Biocoat^™^ Matrigel invasion chamber (BD Bioscience, San Jose, CA). Equal number of transfected cells in serum-free media were added into the upper chamber. The invasion was triggered by adding the same medium supplemented with 10% FBS to the bottom chambers as a chemoattractant. After incubation for 24 h, the invaded cells were fixed with 95% MeOH for 5 min and stained with 0.1% hematoxylin and eosin. Invasiveness was determined by counting the number of invaded cells in at least ten randomly selected fields. For analysis of cell proliferation rate, the transfected cells were plated in 6-well plates at a density of 5 × 10^4^–1 × 10^5^ cells/well. Cells were trypsinized and the number of viable cells was assessed under a microscope at the indicated time points. For clonogenicity assay, the transfected cells were plated in triplicate in 6-well plates and cultured for 2 weeks. Cells were fixed with 4% paraformaldehyde and stained with 0.2% crystal violet. The stained colonies were counted using the Image J program.

### Cellular fractionation

A cellular fractionation assay was performed to determine the subcellular localization of *LINC00263*^8^. Briefly, HeLa cells were lysed with RSB buffer (10 mM Tris-HCl, pH 7.4, 2.5 mM MgCl_2_, 100 mM NaCl) containing 4 mg/ml digitonin (BN2006, Thermo Fisher Scientific). After centrifugation, the supernatant was collected as the cytosolic extract. The remaining nuclear pellet was washed five times with RSB buffer and then lysed with RIPA buffer. The protein levels of α-tubulin and lamin B served as markers for the cytosolic and nuclear fraction, respectively.

### Ribonucleoprotein immunoprecipitation

The association of hnRNPK with *LINC00263* was assessed by ribonucleoprotein immunoprecipitation (RNP-IP) using a hnRNPK-specific antibody (ChIP grade) as described in our previous report^8^. In case of direct interaction between miRNA and its targets, we used antibody recognizing Argonaute 2 (Ago2). Dynabeads^™^ Protein G (Invitrogen) was coupled with the indicated antibody followed by incubation of cytoplasmic lysate prepared using polysome extraction buffer with the antibody-conjugated beads. Following treatment with DNase I and protease K, RNAs were isolated from beads and the enrichment of target RNA level was determined by RT-qPCR analysis. The level of *18S* was used for normalization in all RNP-IP experiments. Representative results are put into the figures and three independent results are presented in Supplementary figures. Details of the antibodies and primers used are provided in Supplementary Tables 2 and 3.

### Antisense oligonucleotide (ASO) pull-down assay

To identify LINC00263-associated miRNAs, ASO pull-down was performed using nonoverlapping biotinylated ASOs recognizing *LacZ* (four ASOs) and *LINC00263* (eight ASOs). Incubation of the whole-cell lysates with the biotinylated ASO was followed by coupling with Streptavidin-coupled Dynabeads^™^ (Invitrogen). RNAs were isolated from the pull-down materials and small RNA sequencing was performed.

### Stoichiometric quantitation of *LINC00263* and miR-147a by RT-qPCR and droplet digital PCR analysis (ddPCR)

We employed ddPCR to quantify the absolute RNA copy numbers of *LINC00263* and the miR-147a in HeLa cells. Briefly, 1.875 μg of total RNA was reversely transcribed with either a First-Strand cDNA synthesis kit or a Mir-X miRNA First-Strand Synthesis kit. The PCR reaction using the droplets was generated using EvaGreen Supermix (1864033, Bio-Rad) containing 1 μl of cDNA for quantifying the copy numbers of *LINC00263* and miR-147a. In both cases, we employed 250 nM primers of the respective RNA. The droplets were generated using the QX200^™^ AutoDG^™^ Droplet Digital^™^ PCR System. The PCR amplification setup was: 5 min at 95 °C, 30 s at 95 °C followed by 60 s at 60 °C for 40 cycles, 5 min at 95 °C, and then held at 4 °C. The absolute RNA copy numbers were assessed using QX200 Droplet Digital PCR System and calculated as described^13^. We further estimated the copy numbers per cell using a reference mRNA of known abundance as described^14^. Following primer sets were used: (F) ATTGGCAAGATGTTCCTTGC and (R) CAAAGCCTGTTTGGTGGTTT for *LINC00263*¸ and GTGTGTGGAAATGCTTCTGC for miR-147a.

### Luciferase reporter assay

To verify the direct interaction between miR-147a and MRE in its target, pmirGLO dual-luciferase vectors (E133A, Promega, Madison, WI) containing wild-type or mutant MRE sequences from *LINC00263* or *CAPN2* mRNA were constructed. Following transfection with control or pre-miR-147a, an equal number of HeLa cells were plated into 24-well plates. Then the cells were transfected with either wild-type or mutant luciferase vector. Luciferase expression was assessed using a Dual-GLO^™^ Luciferase Assay System (E2940, Promega).

## Results

### hnRNPK is responsible for the malignant phenotypes of cancer cells

**Fig. 1:**
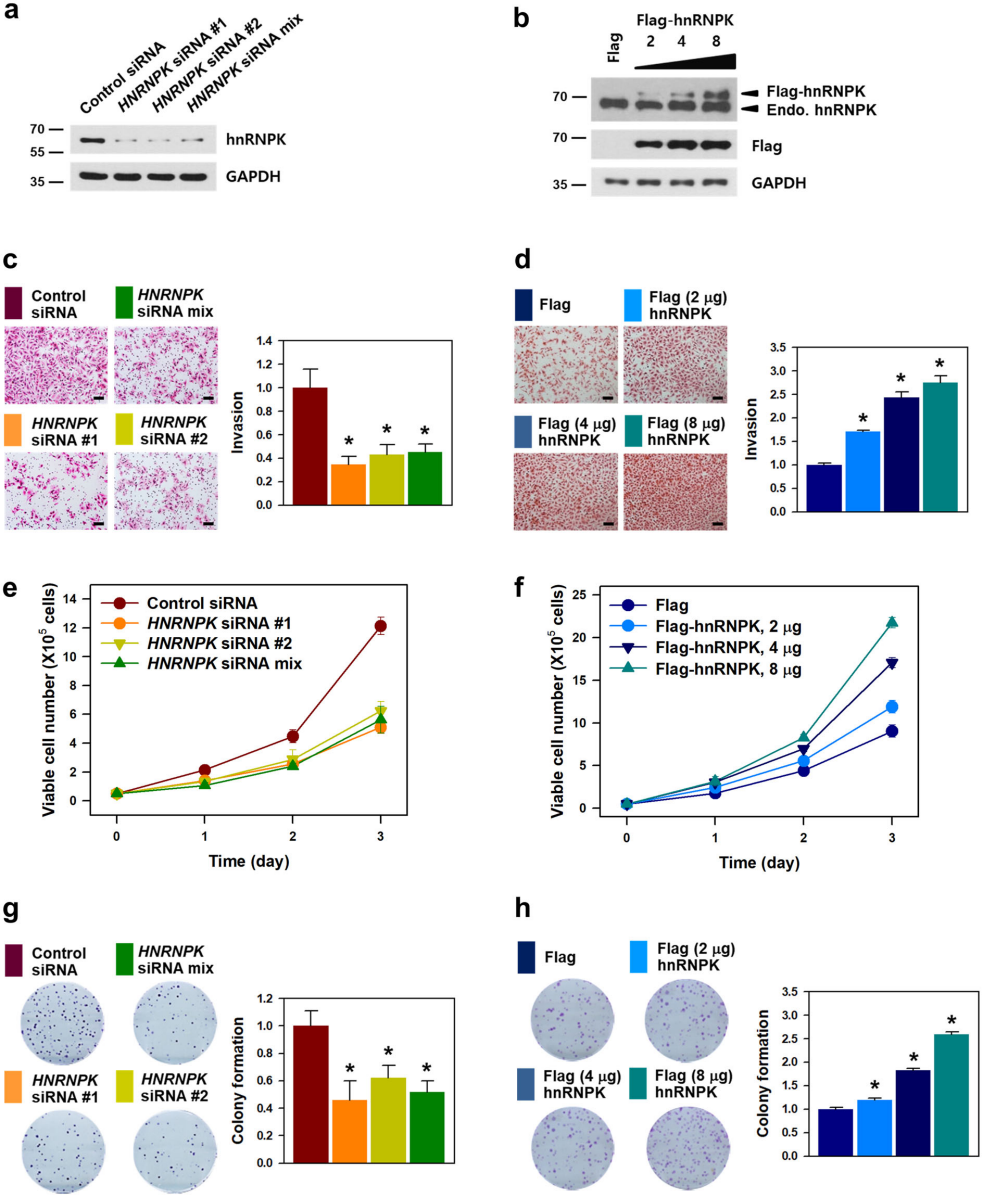
hnRNPK is responsible for the cancer malignancy. To investigate the role of hnRNPK in malignant phenotype, two individual and mixed *HNRNPK* siRNAs were transfected into HeLa cells (**a**, **c**, **e**, and **g**). In contrast, the Flag-hnRNPK vector was constructed and used for hnRNPK overexpression (**b**, **d**, **f**, and **h**). **a**, **b** The efficiency of hnRNPK knockdown (**a**) and overexpression (**b**) was determined by analyzing the level of hnRNPK by Western blot analysis. **c**, **d** Invasive ability was assessed by Transwell invasion assay in vitro. Representative images of the invaded cells are shown. Invasiveness was determined by counting the number of invaded cells from more than ten fields. **e**, **f** To determine the proliferation rate, an equal number of transfected HeLa cells were plated in 12-well plates and the number of viable cells was counted under a microscope at the indicated times. **g**, **h** Equal number of transfected cells were plated in six-well plates, and clonogenicity was determined by counting the number of colonies. Bars on microscopic images represent 100 μm. Statistical analyses were performed using the Student’s *t*-test using three independent experiments (*p < 0.05). All data represent mean ± standard variation (SD).

We investigated the role of hnRNPK in malignant phenotypes including metastatic potential and proliferation in HeLa cells. Individual and mixture of *HNRNPK* siRNAs efficiently decreased hnRNPK expression (Fig. 1a). Conversely, the introduction of Flag-hnRNPK^8^ resulted in a significant increase of hnRNPK in a dose-dependent manner (Fig. 1b). The invasive ability was reduced by hnRNPK knockdown (Fig. 1c); conversely, overexpression of hnRNPK enhanced the invasive ability (Fig. 1d), indicating that hnRNPK is closely associated with the invasiveness of cancer cells.

Two other distinctive features of malignancy, the proliferation rate, and clonogenicity were also examined. Knockdown of hnRNPK resulted in a decrease in the proliferation rate (Fig. 1e). On the other hand, hnRNPK-overexpressing cells showed a higher proliferation rate than the blank vector control cells (Fig. 1f). Knockdown of hnRNPK abrogated the colony-forming ability (Fig. 1g). In contrast, the number of colonies was dose-dependently increased following hnRNPK overexpression (Fig. 1h). Collectively, our findings demonstrate that hnRNPK is responsible for the malignant characteristics including high invasiveness and rapid proliferation.

### *LINC00263* is identified as a novel hnRNPK-regulated lincRNA

**Fig. 2:**
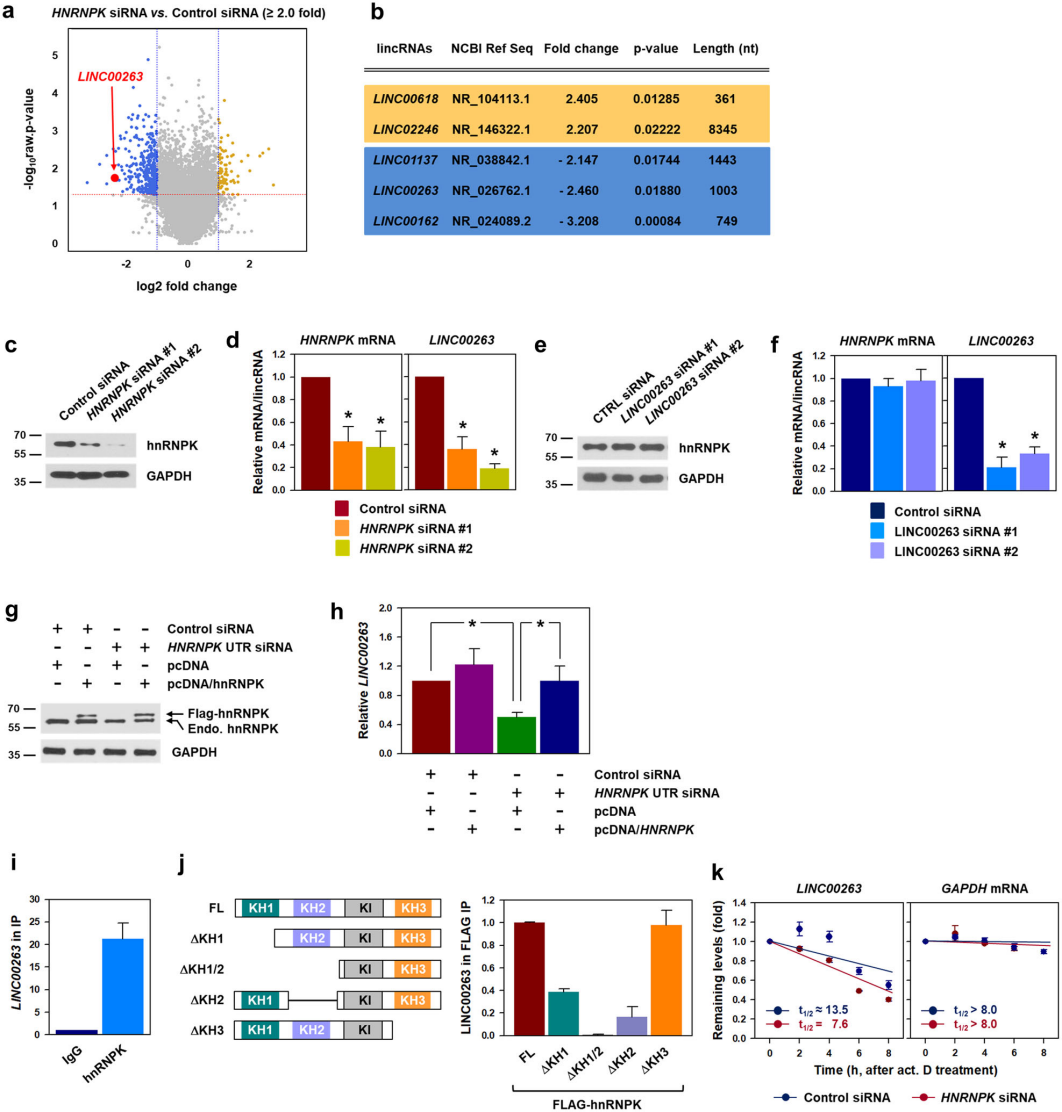
*LINC00263* is a novel target of hnRNPK. **a**, **b** To identify hnRNPK-regulated lincRNAs, RNA sequencing was performed using total RNAs isolated from hnRNPK- and *LINC00263*-silenced HeLa cells. **a** Volcano plots were generated by analyzing the sequencing data. **b** Information about the five hnRNPK-regulated lincRNAs is summarized. **c**, **d** RNA sequencing results were verified by assessing the level of *LINC00263* in hnRNPK-silenced cells. Knockdown of hnRNPK by two independent siRNAs was verified by Western blot analysis (**c**) and the levels of *HNRNPK* mRNA and *LINC00263* were determined by RT-qPCR analysis (**d**). To check whether knockdown of *LINC00263* affects hnRNPK expression, the levels of hnRNPK protein (**e**) and *HNRNPK* mRNA (**f**) were determined by Western blot and RT-qPCR analyses, respectively. **g**, **h** To confirm that hnRNPK regulates *LINC00263*, a specific siRNA targeting the 3′UTR of *HNRNPK* mRNA was used. HeLa cells were cotransfected with the 3′UTR-specific *HNRNPK* siRNAs and Flag-hnRNPK vector. The level of endogenous and ectopic hnRNPK (Flag-hnRNPK) was determined by Western blot analysis. GAPDH was used as a loading control (**g**). The level of *LINC00263* in transfected cells as described above was determined by RT-qPCR analysis (**h**). **i** Direct association of hnRNPK with *LINC00263* was tested by RNP-IP experiment using control IgG and hnRNPK antibody. The enrichment of *LINC00263* was calculated by comparing the level of *LINC00263* in IgG and hnRNPK IP materials. The level of LINC00263 was determined by RT-qPCR analysis and 18S was used for normalization. **j** Following transfection of HeLa cells with wild-type (full length, FL) or four deletion mutant vectors, RNP-IP was performed using an anti-Flag antibody. The level of *LINC00263* in the Flag IP was quantified by RT-qPCR analysis. The Schematic represents wild-type and four mutants of hnRNPK (ΔKH1, ΔKH1/2, ΔKH2, and ΔKH3) used in this study. **k** The effect of hnRNPK on the stability of LINC00263 was examined. Following treatment of actinomycin D (0.5 μg/ml), cells were harvested at the indicated times and the levels of *LINC00263* and *GAPDH* mRNA were determined by RT-qPCR analysis. Statistical analyses were performed using the Student’s *t*-test using three independent experiments (*p < 0.05). All data represent mean ± standard variation (SD).

To identify hnRNPK-regulated lincRNAs, we performed RNA sequencing using hnRNPK-silenced HeLa cells (whole sequencing data were shown in Supplementary materials). The various plots representing RNA sequencing data and gene ontology (GO) analysis are shown in Supplementary Fig. 1a, c. Based on the data analysis and processing, five lincRNAs were identified to be significantly regulated by hnRNPK: two lincRNAs were upregulated and three lincRNAs were downregulated (Fig. 2a, b). Since *LINC00263* showed the most significant effect on the metastatic potential (data not shown), we chose to investigate its role in the control of cancer malignancy through hnRNPK.

To verify the RNA sequencing data, we performed transient knockdown of hnRNPK and observed a substantial decrease in hnRNPK expression with two individual siRNAs (Fig. 2c). Further, knockdown of hnRNPK also reduced the level of *LINC00263* significantly (Fig. 2d). Although *LINC00263*-targeting siRNAs caused a substantial decrease in *LINC00263* level, they did not affect the levels of hnRNPK protein and mRNA (Fig. 2e, f, respectively). In addition, knockdown of hnRNPK by 3′UTR-targeting siRNA efficiently decreased the expression of hnRNPK without significant change in the ectopic hnRNPK (Flag-hnRNPK) (Fig. 2g) and resulted in decreased expression of *LINC00263*. However, the level of *LINC00263* was restored to the control level following the ectopic expression of hnRNPK (Fig. 2h).

Next, we investigated the detailed molecular mechanism by which hnRNPK regulates the expression of *LINC00263*. Since five hnRNPK motives are predicted in the sequence of *LINC00263* (http://rbpmap.technion.ac.il/) (Supplementary Fig. 2a, b), the direct interaction between hnRNPK and *LINC00263* was examined through the RNP-IP experiment. *LINC00263* was found to be highly enriched in hnRNPK IP material compare to control IgG (Fig. 2i). In addition, RNP-IP using full-length Flag-hnRNPK and its various deletion mutants (ΔKH1, ΔKH1/2, ΔKH2, and ΔKH3) revealed that interaction of hnRNPK with *LINC00263* was dependent on its K homology 1 (KH1) and KH2 domains (Fig. 2j). We also examined whether hnRNPK influences the stability of *LINC00263* (Fig. 2k). Knockdown of hnRNPK induced a more rapid decrease in *LINC00263* to compare to control. However, the level of *GAPDH* mRNA was barely affected by the knockdown of hnRNPK.

### *LINC00263* promotes malignant phenotypes including invasiveness, proliferation, and clonogenicity

**Fig. 3:**
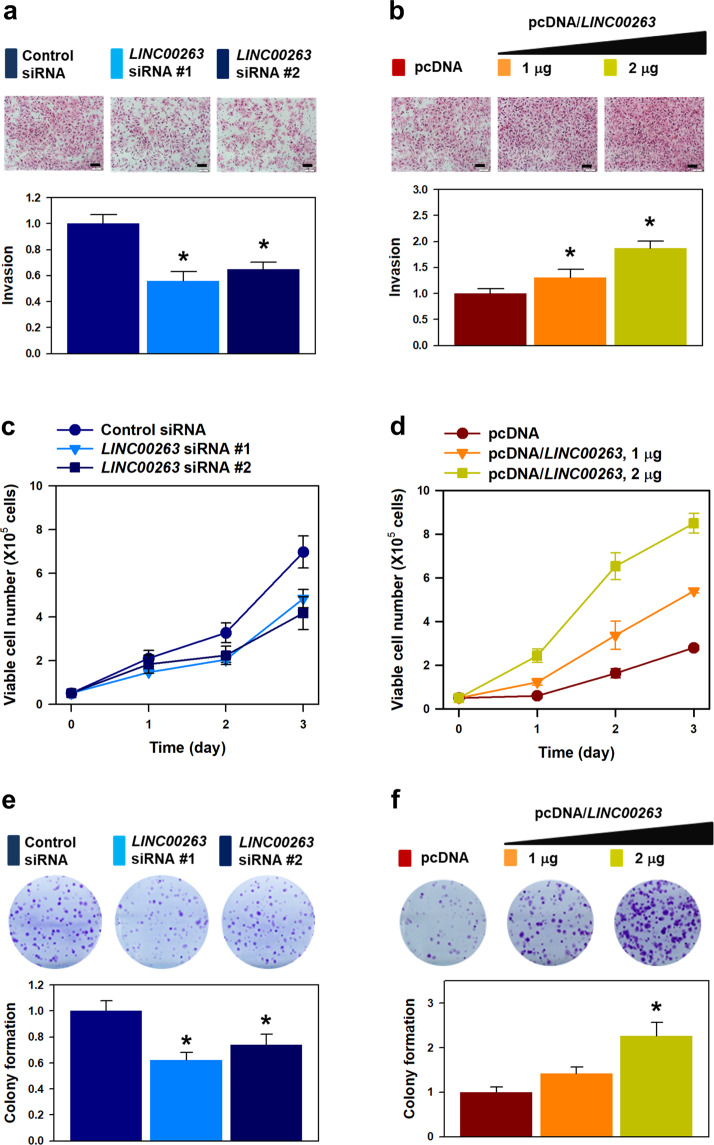
*LINC00263* regulates the malignant phenotypes. To investigate whether *LINC00263* is required for hnRNPK-mediated malignant phenotypes, two independent siRNAs (**a**, **c**, and **e**) and overexpression vector (pcDNA/*LINC00263*: **b**, **d**, and **f**) were used. Invasiveness was determined using Transwell invasion assay (**a**, **b**). Cellular proliferation (**c**, **d**) and clonogenicity (**e**, **f**) were assessed by counting the number of viable cells and colonies, respectively, as described in “Materials and methods”. Bars on microscopic images represent 100 μm. Statistical analyses were performed using the Student’s *t* test using three independent experiments (*p < 0.05). All data represent mean ± standard variation (SD).

To investigate whether *LINC00263* is responsible for hnRNPK-mediated invasiveness, we assessed invasive ability. Knockdown of *LINC00263* in HeLa cells showed a decrease in the number of invading cells (Fig. 3a). Conversely, overexpression of *LINC00263* potentiated the invasive ability of HeLa cells (Fig. 3b). The level of *LINC00263* in the overexpressing cells was verified by RT-qPCR analysis (Supplementary Fig. 3a). We also observed decreased proliferation rate in *LINC00263*-silenced cells (Fig. 3c). In contrast, the proliferation rate tended to increase in *LINC00263*-overexpressing cells (Fig. 3d). The colony-forming assay revealed that approximately 40% decrease in the number of colonies in *LINC00263*-silenced cells (Fig. 3e), while the colony-forming ability was increased following overexpression of *LINC00263* (Fig. 3f). These results indicate that *LINC00263* is associated with the oncogenic function of hnRNPK.

### miR-147a is involved in the regulation of cancer malignancy by hnRNPK/*LINC00263*

**Fig. 4:**
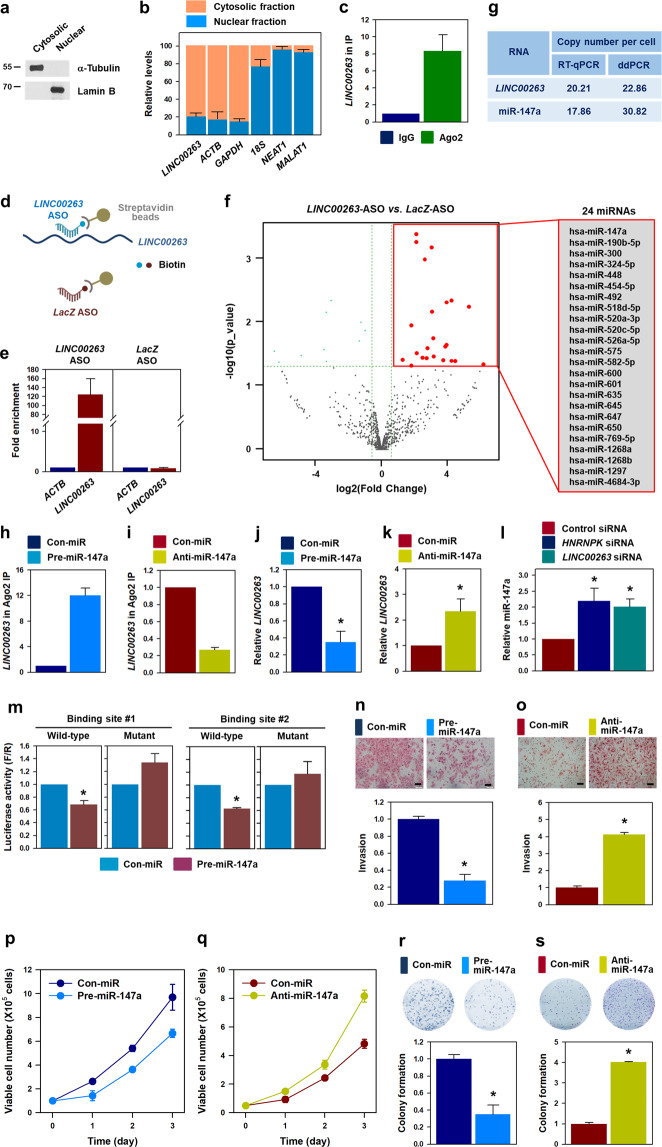
*LINC00263* functions as ceRNA for miR-147a. **a**, **b** Cellular fractionation assay was performed to check the localization of *LINC00263*. To ensure the purity of the fractions, the levels of α-tubulin (cytosolic marker) and lamin B (nuclear marker) were analyzed by Western blot analysis (**a**). The levels of *LINC00263*, *18S*, *GAPDH*, and *ACTB* mRNA in each fraction were determined by RT-qPCR analysis (**b**). **c** To check whether *LINC00263* is involved in miRISC, Ago2 RNP-IP was performed using a specific antibody. The level of *LINC00263* in control IgG and Ago2 IP materials was determined by RT-qPCR analysis and normalized to the level of *GAPDH* mRNA. **d**–**f** To screen for *LINC00263*-associated miRNAs, antisense oligonucleotide pull-down (ASO PD) was performed. A schematic of the experimental design is shown (**d**). Detailed information of the ASO sequences for *LacZ* (control) and *LINC00263* was provided in Supplementary Fig. 4a. The efficiency of ASO PD was examined by comparing the level of *LINC00263* in ASO PD materials (**e**). Small RNA sequencing was performed with RNAs isolated from ASO PD materials. miRNAs with higher expression in *LINC00263* ASO PD are listed (**f** and Supplementary fig. 4b). **g** Quantification of copy numbers of LINC00263 and miR-147a (copy number per cell) was performed by RT-qPCR and ddPCR analyses. **h**–**k** HeLa cells were transfected with pre-miR-147a (for overexpression, **h** and **j**) or anti-miR-147a (for inhibition, **i** and **k**). Direct association of *LINC00263* with miR-147a-involved miRISC was analyzed by Ago2 RNP-IP (**h**, **i**) and the level of *LINC00263* was determined by RT-qPCR analysis (**j**, **k**). **l** Following the knockdown of hnRNPK and *LINC00263*, the level of miR-147a was determined by RT-qPCR analysis. **m** Bioinformatic analyses revealed that two MREs of miR-147a exist in the *LINC00263* sequence (Supplementary Fig. 7a, b). To examine the sequence-specific interaction, luciferase reporter vectors containing wild-type or mutant sequences of miR-147a MREs were constructed. Following overexpression of miR-147a, the luciferase activity was assessed as described in “Materials and methods”. **n**–**s** To investigate the effect of miR-147a on malignant capabilities, pre-miR-147a (**n**, **p**, and **r**) or anti-miR-147a (**o**, **q**, and **s**) were introduced into HeLa cells. Malignant phenotypes including invasiveness (**n**, **o**), proliferation rate (**p**, **q**), and clonogenicity (**r**, **s**) were examined as described in “Materials and methods”. Bars on microscopic images represent 100 μm. Statistical analyses were performed using the Student’s *t* test using three independent experiments (*p < 0.05). All data represent mean ± standard variation (SD).

To determine the molecular mechanism through which *LINC00263* positively regulates malignant properties, we first examined the subcellular localization of *LINC00263*. For verification of the appropriate cellular fractions, the levels of α-tubulin (cytosolic marker) and lamin B (nuclear marker) were analyzed in each fraction (Fig. 4a). The levels of *NEAT1*, *MALAT1*, *18S*, *GAPDH*, and *ACTB* were assessed for reference (Fig. 4b). The cellular fractionation assay revealed that *LINC00263* was mainly localized in the cytosol, whereas *NEAT1* and *MALAT1* were dominantly expressed in the nucleus. In addition, we performed Argonaute 2 immunoprecipitation (Ago2-IP) assay to examine whether *LINC00263* was associated with the function of miRNAs (Fig. 4c). *LINC00263* was more enriched in Ago2-IP compared to control IgG-IP, indicating that *LINC00263* is involved in the regulatory pathway of miRNAs (Supplementary Fig. 5a).

From the above results, we hypothesized that *LINC00263* may function as a competitive endogenous RNA (ceRNA) for tumor-suppressing miRNA. To identify *LINC00263*-associated miRNAs, ASOs for *LINC00263* and *LacZ* were designed to perform ASO pull-down experiments (Fig. 4d and Supplementary Fig. 4a). The efficiency of the ASO pull-down was verified by determining the levels of *LINC00263* and *ACTB* mRNA in ASO pull-down materials. Whereas *ACTB* mRNA was not enriched, *LINC00263* was selectively enriched in the pull-down materials using the corresponding ASOs as compared to *LacZ* ASO (Fig. 4e). To screen *LINC00263*-bound miRNAs, small RNA sequencing was performed using the RNA isolated from the ASO pull-down. Analysis of sequencing data revealed that 24 miRNAs showed higher enrichment in *LINC00263* ASO pull-down material than in *LacZ* ASO pull-down (Fig. 4f and Supplementary Fig. 4b). Next, we predicted the potential miRNA binding sites within the *LINC00263* sequence using a miRNA target discovery tool RNA22 (https://cm.jefferson.edu/rna22). This bioinformatics tool revealed that *LINC00263* possessed MREs for only four miRNAs (miR-147a, miR-492, miR-601, and miR-1268a) out of the 24 miRNAs found by the ASO pull-down analyses (Supplementary Fig. 4c). Since miR-147a showed the most significant folding energy, we chose to further investigate whether miR-147a was responsible for the oncogenic function of hnRNPK/*LINC00263*.

Under basal conditions, *LINC00263* was present in 20–23 copies per cell, whereas miR-147a was present in 17–31 copies per cell as assessed by RT-qPCR and ddPCR experiments (Fig. 4g). The fact that the copy numbers are comparable in cells lends support to their stoichiometric interaction. In addition, miR-147a was found to be highly enriched in *LINC00263* ASO pull-down materials (Supplementary Fig. 4d). Ago2-IP assay indicated that overexpression of miR-147a resulted in an increase in *LINC00263* in Ago2 IP materials, indicating that miR-147a guided the interaction of *LINC00263* with Ago2 to form miRNA-induced silencing complex (miRISC) (Fig. 4h). In contrast, inhibition of miR-147a using anti-miR-147a decreased the level of *LINC00263* in Ago2-IP (Fig. 4i). Pre- and anti-miR-147a significantly increased and decreased the level of miR-147a (Supplementary Fig. 3b, c). These results indicate that *LINC00263* is associated with miR-147a-guided miRISC. Decreased level of *LINC00263* was observed in miR-147a-overexpressing cells compared to that in the control (Fig. 4j); conversely, *LINC00263* was highly expressed following miR-147a knockdown (Fig. 4k). Accordingly, we assessed the level of miR-147a in hnRNPK- and *LINC00263*-silenced cells (Fig. 4l). Knockdown of both hnRNPK and *LINC00263* resulted in an increase of miR-147a, indicating that *LINC00263* acts as a ceRNA for miR-147a. Two MREs of miR-147a in *LINC00263* were predicted by the bioinformatic tool (Supplementary Fig. 7a, b). Consequently, we constructed luciferase reporter vectors containing wild-type or mutant sequence of miR-147a MREs. In both reporter vectors, overexpression of miR-147a suppressed the expression of luciferase in the wild-type reporter vector but not in the mutant (Fig. 4m). The results of the Ago2-IP and luciferase reporter assay revealed that miR-147a directly binds to *LINC00263*.

Next, we tested whether miR-147a influences malignant phenotypes. Invasiveness was reduced by overexpression of miR-147a; conversely, inhibition of miR-147a resulted in increased invasive ability (Fig. 4n, o, respectively). In addition to invasiveness, proliferation rate and colony-forming ability were also regulated by miR-147a. Under conditions of high miR-147a levels, the proliferation rate and clonogenicity were diminished (Fig. 4p, r, respectively). Conversely, a decrease in miR-147a level resulted in higher proliferative and clonogenic abilities compared to those of the control (Fig. 4q, s, respectively). Collectively, we concluded that *LINC00263* controls malignant properties by functioning as a ceRNA of miR-147a.

### CAPN2 is a target of hnRNPK/*LINC00263*/miR-147a axis

**Fig. 5:**
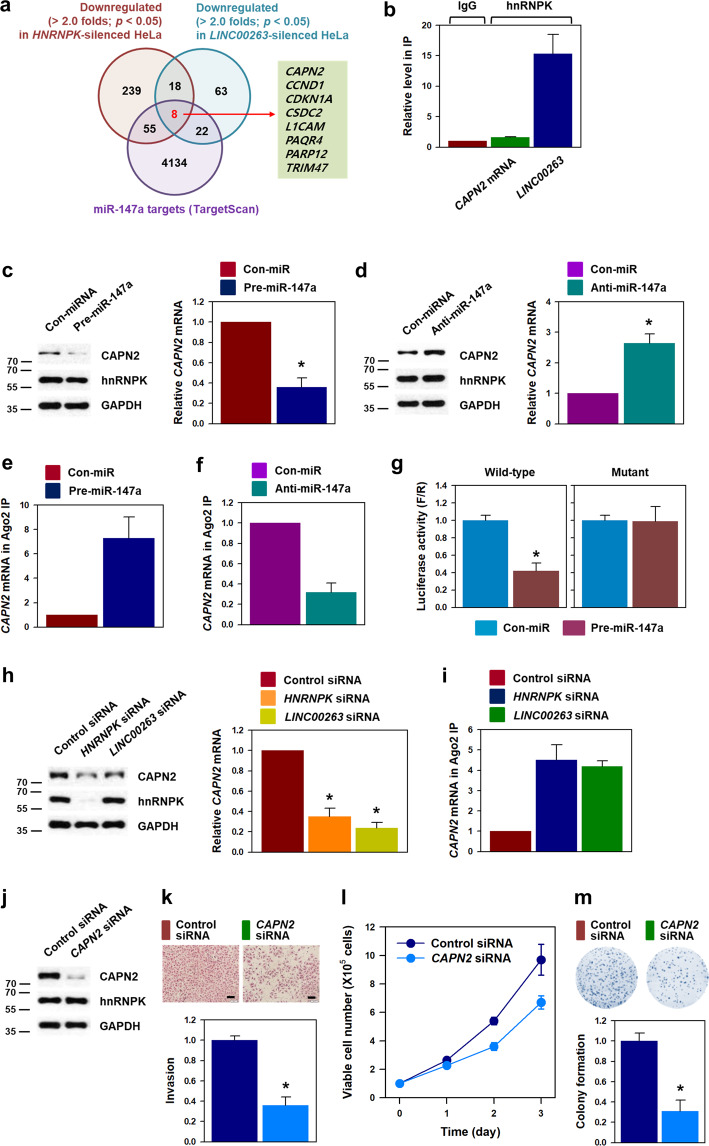
CAPN2 is responsible for the oncogenic function of hnRNPK/*LINC00263*/miR-147a. **a** By comparing RNA sequencing data and miR-147a predicted target genes (TargetScan v7), eight genes were selected as putative targets of hnRNPK/*LINC00263*/miR-147a. **b** To check whether the interaction of hnRNPK with *CAPN2* mRNA is required for the regulation of its expression, an RNP-IP experiment was performed. The levels of *CAPN2* mRNA and *LINC00263* in each IP material were determined by RT-qPCR analysis. **c**–**g** Following transfection of HeLa cells with pre-miR-147a (for overexpression, **c**, **e**, and **g**) or anti-miR-147a (for inhibition, **d** and **f**), the levels of CAPN2 protein and mRNA were determined by Western blot and RT-qPCR analyses, respectively. To examine whether miR-147a directly binds to the 3′UTR of *CAPN2* mRNA, Ago2 RNP-IP (**e**, **f**) and luciferase reporter assay (**g**) were performed. Detailed information of luciferase reporter vector is presented in Supplementary Fig. 7c. **h** Protein and mRNA expression of *CAPN2* in hnRNPK- and *LINC00263*-silenced cells were determined by Western blot and RT-qPCR analyses, respectively. **i** To examine whether knockdown of hnRNPK or *LINC00263* influences the interaction between miR-147a and *CAPN2* mRNA, Ago2 RNP-IP assay was performed as described in “Materials and methods”. **j**–**m** The effect of *CAPN2* silencing on malignant phenotypes including invasiveness (**k**), proliferation (**l**), and clonogenicity (**m**) were investigated. The efficiency of CAPN2 silencing was determined by Western blot analysis (**j**). Bars on microscopic images represent 100 μm. Statistical analyses were performed using the Student’s *t* test using three independent experiments (*p < 0.05). All data represent mean ± standard variation (SD).

To search for target genes responsible for the oncogenic function of hnRNPK/*LINC00263*/miR-147a, we conducted RNA sequencing using total RNA isolated from hnRNPK- and *LINC00263*-silenced HeLa cells (Supplementary Fig. 1a, b). TargetScan (http://www.targetscan.org) was used to predict miR-147a target genes. Eight genes (*CAPN2*, *CCND1*, *CDKN1A*, *CSDC2*, *L1CAM*, *PAQR4*, *PARP12*, and *TRIM47*) were identified as common target genes that are simultaneously regulated by hnRNPK, *LINC00263*, and miR-147a (Fig. 5a). RT-qPCR analysis indicated that knockdown of either hnRNPK or *LINC00263* significantly decreased the level of *CAPN2* mRNA, suggesting that it may be a putative target of hnRNPK/*LINC00263*/miR-147a (Supplementary Fig. 6). Whereas *LINC00263* was highly enriched in hnRNPK IP material, *CAPN2* mRNA was barely bound to hnRNPK (Fig. 5b). Overexpression of miR-147a resulted in decreased *CAPN2* protein expression without any change in hnRNPK (Fig. 5c). Conversely, inhibition of miR-147a resulted in increased CAPN2 protein and mRNA expression (Fig. 5d). To determine the direct binding between *CAPN2* mRNA and miR-147a, the level of *CAPN2* mRNA in Ago2-IP material was assessed. Ago2-IP revealed that miR-147a increased the enrichment of *CAPN2* mRNA in miRISC (Fig. 5e); conversely, knockdown of miR-147a decreased the level of CAPN2 mRNA in the Ago2-IP material (Fig. 5f). In addition to Ago2-IP, luciferase reporter vectors containing the wild-type and mutant MRE of miR-147a were constructed to confirm the direct binding of miR-147a to the 3′UTR of *CAPN2* mRNA. Overexpression of miR-147a inhibited luciferase activity in wild-type vector, whereas it did not affect the expression of luciferase in mutant vector (Fig. 5g). In addition to luciferase assay, knockdown of hnRNPK and *LINC00263* significantly decreased the level of CAPN2 protein and mRNA (Fig. 5h). *CAPN2* mRNA was enriched in Ago2-IP following knockdown of hnRNPK or *LINC00263* (Fig. 5i), suggesting that a decrease in hnRNPK strengthens the function of miR-147a by reducing *LINC00263*.

To validate whether CAPN2 is involved in the regulation of malignant phenotypes by hnRNPK/*LINC00263*/miR-147a, the effect of CAPN2 silencing on invasiveness, proliferation, and clonogenicity was examined. Introduction of *CAPN2*-specific siRNA into HeLa cells markedly decreased CAPN2 expression (Fig. 5j). As expected, knockdown of CAPN2 decreased the number of invading cells (Fig. 5k), inhibited cell proliferation (Fig. 5l), and suppressed colony-forming ability (Fig. 5m).

In the proteome profiler human p-kinase array, phosphorylation of ERK and p70S6K was found to be diminished in *HNRNPK*- or *LINC00263*-silenced cells compared to the controls (Supplementary Fig. 8a, b). Western blot analysis verified that knockdown of hnRNPK or *LINC00263* reduced phosphorylated ERK and p70S6K. Further, decreased expression of *CAPN2* using miR-147a or siRNA inhibits the activation of ERK and p70S6K (Supplementary Fig. 8c). Collectively, we demonstrated that CAPN2 was responsible for the oncogenic function as a target of hnRNPK/*LINC00263*/miR-147a and that ERK and p70S6K pathways are partly involved.

### Repression of malignant capabilities is restored by miR-147a inhibition or CAPN2 overexpression

**Fig. 6:**
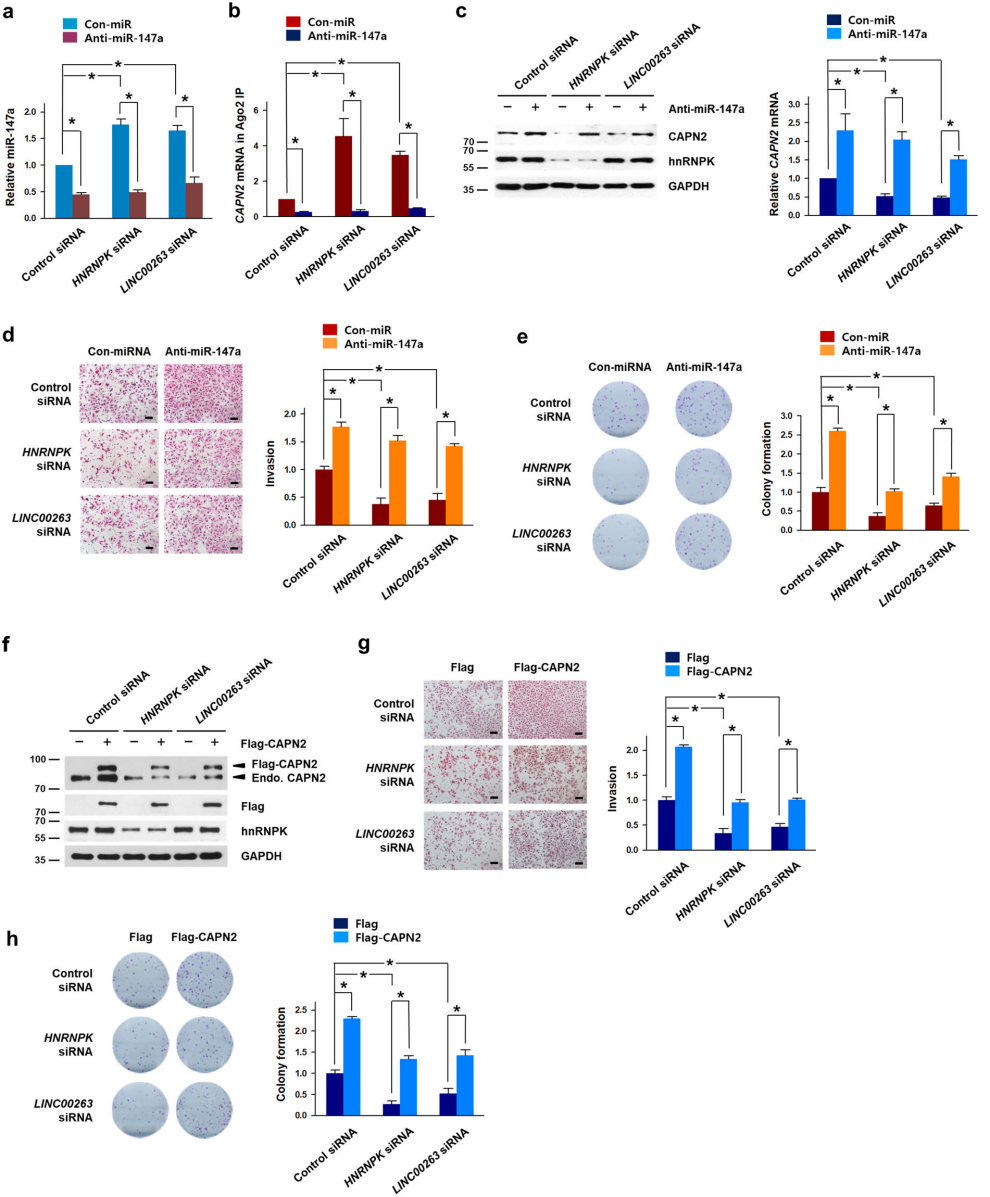
Repression of malignant phenotypes following knockdown of hnRNPK and *LINC00263* is restored by inhibition of miR-147a or ectopic expression of CAPN2. **a**–**e** To examine whether inhibition of miR-147a restores the malignant capabilities, siRNAs for hnRNPK or *LINC00263* were introduced into HeLa cells with control miRNA or anti-miR-147a. Following isolation of total RNA, the level of miR-147a was determined by RT-qPCR analysis (**a**). Ago2 RNP-IP experiment was performed using the cytoplasmic lysates. The level of *CAPN2* mRNA in Ago2 IP material was determined by RT-qPCR analysis (**b**). The expression levels of CAPN2 protein and mRNA were determined by Western blot and RT-qPCR analyses, respectively (**c**). Invasiveness (**d**) and colony-forming ability (**e**) were examined as described in “Materials and methods”. **f**–**h** For the rescue experiments, CAPN2 was ectopically overexpressed in hnRNPK- or *LINC00263*-silenced HeLa cells. The protein level of CAPN2 was determined by Western blot analysis (**f**). Invasiveness (**g**) and colony-forming ability (**h**) were examined as described in “Materials and methods”. Bars on microscopic images represent 100 μm. Statistical analyses were performed using the Student’s *t* test using three independent experiments (*p < 0.05). All data represent mean ± standard variation (SD).

From the above results, we found that hnRNPK-regulated *LINC00263* decoys miR-147a and thus increases CAPN2 expression. To verify our findings, we performed rescue experiments by downregulating miR147-a. The level of miR-147a was significantly decreased not only in the control but also in hnRNPK- or *LINC00263*-silenced cells by anti-miR-147a where miR-147a is upregulated by lowering its ceRNA, *LINC00263* (Fig. 6a). Whereas knockdown of hnRNPK or *LINC00263* increased the level of *CAPN2* mRNA in Ago2 IP, the inhibition of miR-147a by anti-miRNA lowered the enrichment of *CAPN2* mRNA in miRISC, indicating that miR-147a is responsible for the repression of CAPN2 in hnRNPK- and *LINC00263*-silenced cells (Fig. 6b). Inhibition of miR-147a reversed the decrease in CAPN2 protein and mRNA caused by the knockdown of hnRNPK and *LINC00263* as well (Fig. 6c). Consistent with the recovery of reduced CAPN2 expression, invasiveness, and colony-forming abilities were restored by anti-miR-147a (Fig. 6d, e, respectively). These results demonstrate that miR-147a is closely involved in the regulation of CAPN2 expression, and thus plays an important role in the gain of malignant phenotypes by hnRNPK/*LINC00263*.

In addition to inhibition of miR-147a, we examined whether the ectopic expression of CAPN2 reverses the lowered malignant capabilities resulting from the knockdown of hnRNPK and *LINC00263*. The appropriate concentration of overexpression vector was determined by introducing various concentrations of Flag-CAPN2 vector (Supplementary Fig. 3d). Western blot analysis showed that knockdown of hnRNPK and *LINC00263* decreased CAPN2 expression and that ectopic CAPN2 did not affect the expression of hnRNPK (Fig. 6f). As observed earlier, invasiveness was significantly decreased following the knockdown of hnRNPK and *LINC00263*. However, following ectopic overexpression of CAPN2, the invasive ability was restored (Fig. 6g). Consistent with the results of the invasion assay, the colony-forming assay revealed that ectopic CAPN2 restored the clonogenic ability that was reduced in the hnRNPK- and *LINC00263*-silenced cells (Fig. 6h). Collectively, we concluded that CAPN2 is a major effector of the oncogenic function of hnRNPK/*LINC00263*/miR-147a.

### The ability to sponge miR-147a is required for the oncogenic potential of *LINC00263*

**Fig. 7:**
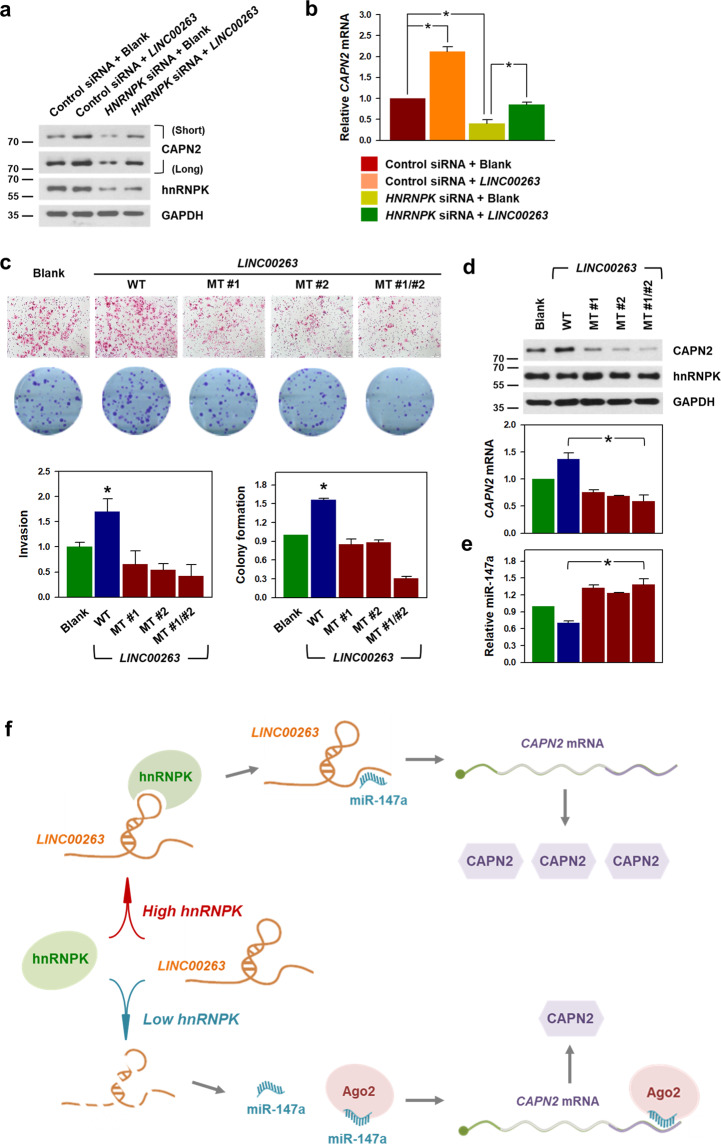
The ability to sponge miR-147a is required for the oncogenic function of *LINC00263*. **a**, **b** HeLa cells were transfected with HNRNPK siRNA and/or a *LINC00263* expression vector. The expression levels of CAPN2 protein (**a**) and CAPN2 mRNA (**b**) were determined by Western blot and RT-qPCR analyses, respectively. **c**–**e** *LINC00263* expression vectors harboring mutant sequences of both miR-147a MREs were constructed. As with constructing the luciferase reporter vectors, four nucleotides of each miR-147a MRE in *LINC00263* were changed to block the binding of miR-147a. **c** The invasive and clonogenic effects of three mutated *LINC00263* (miR-147a MRE mutant #1, #2, and #1/#2) were determined as described in “Material and methods”. **d** The expression levels of CAPN2 protein and CAPN2 mRNA were determined by Western blot and RT-qPCR analyses, respectively. **e** Effect of mutated *LINC00263* on miR-147a expression was assessed by RT-qPCR. Statistical analyses were performed using the Student’s *t* test using three independent experiments (*p < 0.05). All data represent mean ± standard variation (SD). **f** Schematic of the proposed mechanism of action of the oncogenic hnRNPK/*LINC00263*/miR-147a/CAPN2-axis. A detailed description is shown in the main text.

To test whether hnRNPK is required for the regulation of CAPN2 expression by *LINC00263*, the expression levels of CAPN2 protein and mRNA were examined in the conditions of hnRNPK presence or absence (Fig. 7a, b). Overexpression of *LINC00263* showed an increase in the expression of CAPN2, regardless of the level of hnRNPK. These results suggest that the control of CAPN2 by *LINC00263* resulted from inhibiting the function of miR-147a.

In addition, we newly constructed *LINC00263* expression vector harboring mutant sequences of two miR-147a MREs to block the binding of miR-147a (detailed information in Supplementary Fig. 10). As observed earlier, *LINC00263* enhanced the invasive ability and increased the number of colonies (Fig. 7c). However, the mutants of *LINC00263* did not show an increase in invasive and clonogenic abilities. Rather, they showed some inhibitory effects (Fig. 7d). It is assumed that ectopic expression of mutated *LINC00263* possibly interrupts the interaction between endogenous *LINC00263* and miR-147a, thus resulting in an increasing amount of working miR-147a (Fig. 7e). Collectively, we concluded that the decoying ability for miR-147a is responsible for the oncogenic functions of *LINC00263*.

Based on our findings, the regulatory role of hnRNPK/*LINC00263*/miR-147a/CAPN2 in cancer malignancy is schematically summarized in Fig. 7f. Briefly, *LINC00263* is regulated by hnRNPK and functions as a ceRNA for *CAPN2*-targeting miR-147a. Under conditions of high hnRNPK, *LINC00263* is highly expressed thereby reducing the amount of *CAPN2*-targeting miR-147a. Conversely, low hnRNPK results in the decreased *LINC00263*, which potentiates miR-147a-mediated suppression of CAPN2. Therefore, the malignant capabilities are diminished. Taken together, our data suggest that hnRNPK/*LINC00263*/miR-147a/CAPN2 represents a promising target for the development of cancer therapeutics.

### hnRNPK/*LINC00263*/miR-147a/CAPN2 axis is applicable to various types of cancer cells

**Fig. 8:**
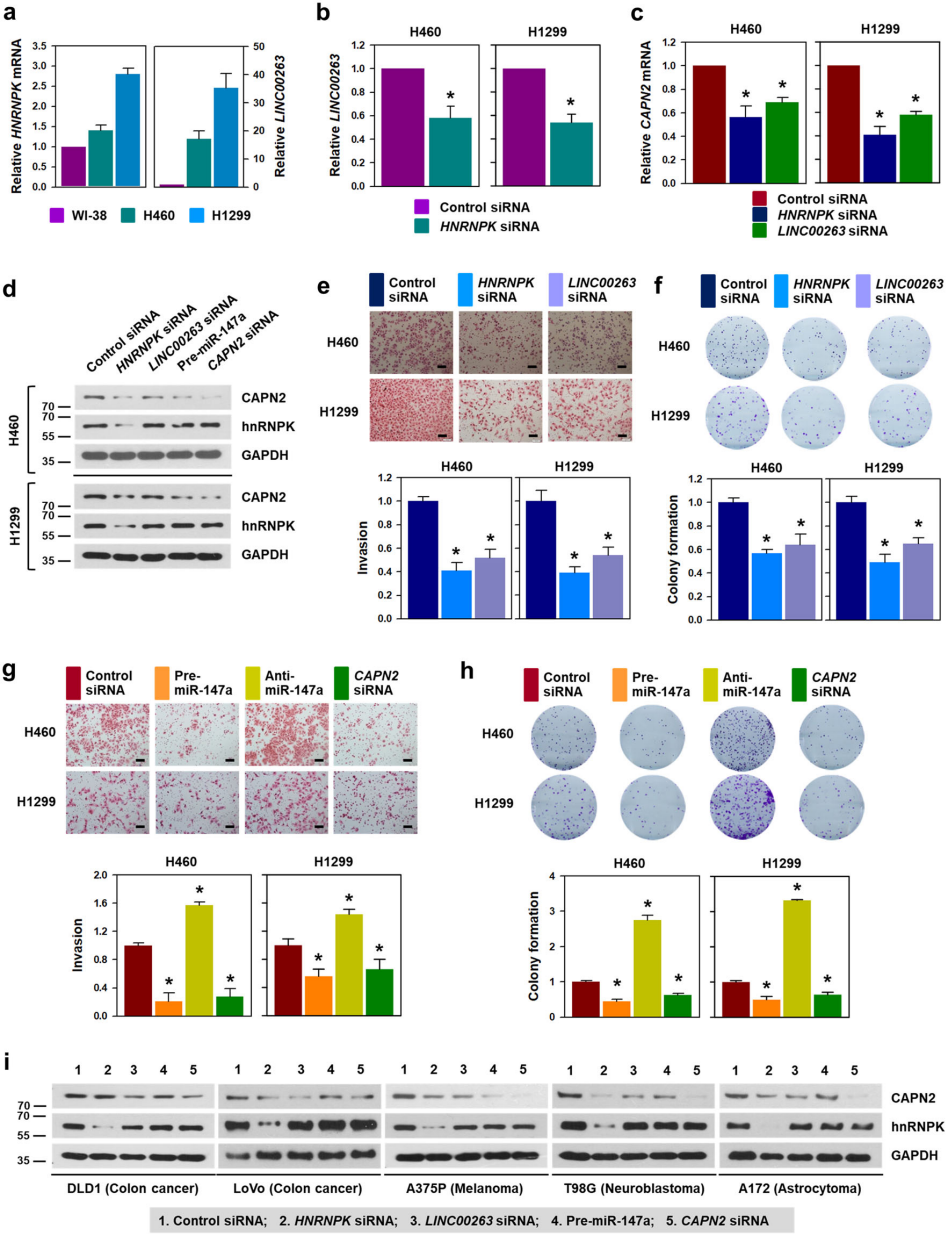
hnRNPK/*LINC00263*/miR-147a/CAPN2 is a promising target for the development of cancer therapeutics. To expand our findings to various types of cancer, we checked whether the regulatory mechanism of hnRNPK/*LINC00263*/miR-147a/CAPN2 is applicable to various types of cancers. **a** The relationship between the expression of *HNRNPK* mRNA and *LINC00263* was examined in lung cancer cells by comparing them to the levels in WI-38 cells. The levels of *HNRNPK* mRNA and *LINC00263* in WI-38, H460, and H1299 were determined by RT-qPCR analysis. **b** To determine whether hnRNPK regulates *LINC00263*, the level of *LINC00263* was analyzed by RT-qPCR analysis in hnRNPK-silenced lung cancer cells. **c** Regulation of CAPN2 by hnRNPK and *LINC00263* was verified by assessing the level of *CAPN2* mRNA in hnRNPK- or *LINC00263*-silenced lung cancer cells. **d**–**h** To examine whether hnRNPK/*LINC00263*/miR-147a/CAPN2 axis regulates the invasive and clonogenic abilities, H460 and H1299 cells were transfected with siRNA targeting *HNRNPK* mRNA or *LINC00263*, or pre-miR-147a. The level of CAPN2 protein was determined by Western blot analysis (**d**). Invasiveness (**e**, **g**) and colony-forming ability (**f**, **h**) were examined as described in “Materials and methods”. **i** The effect of hnRNPK/*LINC00263*/miR-147a on CAPN2 expression was evaluated in various cancer cells including DLD1, LoVo, A375P, T98G, and A172. The expression level of CAPN2 and hnRNPK were determined by Western blot analysis. Bars on microscopic images represent 100 μm. Statistical analyses were performed using the Student’s *t* test using three independent experiments (*p < 0.05). All data represent mean ± standard variation (SD).

To generalize our findings to various types of cancer cells, the regulatory action of hnRNPK/*LINC00263*/miR-147a/CAPN2 was examined in two lung cancer cells (H460 and H1299). We compared the level of *LINC00263* in two GSE datasets (Supplementary Fig. 9a, b)^15^. *LINC00263* was highly expressed in non-small cell lung cancer tissues compared to nonmalignant tissues (GSE81089) and in tumor tissues compared to normal (GSE40419) tissues. In addition, we compared the level of *HNRNPK* mRNA and *LINC00263* in two lung cancer cells with those in non-cancerous WI-38 cells (Fig. 8a). Compared to that in WI-38 cells, the expression of *HNRNPK* and *LINC00263* was significantly increased in both lung cancer cells. Further, the expression level of *HNRNPK* mRNA and *LINC00263* was positively correlated (Fig. 8b). Moreover, H1299 cells showed higher invasive ability than H460 cells (Supplementary Fig. 9c), indicating that the higher the invasiveness, the greater the increase of *HNRNPK* mRNA and *LINC00263*. Consistent with the previous results, knockdown of hnRNPK and *LINC00263* induced a decrease of CAPN2 mRNA in both the lung cancer cells (Fig. 8c). Moreover, the introduction of pre-miR-147a and *CAPN2* siRNA also decreased the expression of CAPN2 (Fig. 8d). As expected, the invasive and clonogenic abilities were diminished following the knockdown of hnRNPK and *LINC00263* (Fig. 8e). We found that *LINC00263* increased the number of invading cells in H460 cells (Supplementary Fig. 9d). The number of colonies was also decreased in *HNRNPK*- and *LINC00263*-silenced cells (Fig. 8f). Overexpression of miR-147a lowered invasive and colony-forming abilities of both the lung cancer cells (Fig. 8g, h, respectively). Conversely, inhibition of miR-147a induced an increase in invasiveness and clonogenicity. From these results, we confirmed that hnRNPK/*LINC00263*/miR-147a/CAPN2 regulatory axis is very closely related to the malignant phenotype of lung cancer cells.

Next, the role of hnRNPK/*LINC00263*/miR-147a in the regulation of CAPN2 expression was verified in various cancer cells including DLD1, LoVo, A375, T98G, and A172 cells (Fig. 8i). All the cells tested showed suppression of CAPN2 expression as observed in HeLa and lung cancer cells. From the above results, we confirmed that our findings are applicable to various types of cancers.

## Discussion

Cancer malignancy, the main cause of high cancer-related mortality, is controlled by strict and precise control of gene expression. Accumulating evidence indicates that RBPs and ncRNAs are key players in post-transcriptional gene regulation by affecting multiple steps of gene expression^16^. RBPs and ncRNAs are also known to modulate multiple cancer traits related to cancer progression, for instance, rapid proliferation and high metastatic potential. HnRNPK is known to be one of the most promising RBP targets for the treatment of various cancers. Typically, hnRNPK accelerates cellular proliferation and potentiates metastatic potential by upregulating a wide range of oncogenes that trigger malignant phenotypes. Recently, the interaction of hnRNPK and ncRNAs, especially lncRNAs, was reported to play a critical role in gene regulation at transcriptional and post-transcriptional levels^17^.

lncRNAs function as critical regulators of cancer metastasis, and their abnormal expression has been reported in many malignant tumors^18,19^. Accumulating evidence suggests that lncRNAs govern many cellular processes related to cancer malignancy partly by associating with various RBPs^20^. Recently, several lncRNAs have been reported to directly interact with hnRNPK in the cytoplasm. The elevated level of hnRNPK-associated lincRNA correlates with metastasis and poor prognosis of cancer patients, for example, c-Myc upregulated lncRNA (*MYU*), cancer susceptibility candidate 11 (*CASC11*), Ets-1 promoter-associated noncoding RNA (*pancEts-1*)^21–24^. In addition, hnRNPK-regulated lincRNAs including *LINC01413* and *LINC00460* potentiates the metastatic potential by inducing epithelial–mesenchymal transition (EMT)^24,25^.

Salmena et al.^10^ established the concept of ceRNA as a group of RNA transcripts that can quantitatively regulate miRNA through the sequence called MRE in ceRNA. Because miRNAs are partially complementary to the 3′UTR of the target mRNA, each miRNA has few hundreds of target genes. So far, several ncRNAs are reported to function as ceRNAs including pseudogenes, antisense transcripts, and lncRNAs^26^. Many ceRNAs are highly expressed in cancer cells compared to normal cells and thus miRNA is more susceptible to degradation in cancer cells. Since the functional efficacy of ceRNA is primarily dependent on its relative level, understanding how ceRNAs are controlled may be useful for the development of cancer treatments. As a ceRNA, lncRNAs compete with miRNAs for binding to their MRE in the endogenous target mRNA, thus causing a reduction and impairment of miRNA. For these reasons, ceRNAs are also termed endogenous miRNA sponges. Several lncRNAs such as *MEG3* and *TUG1*, sequester multiple miRNAs from their target mRNA, thus leading to the derepression of target genes^27–30^. In addition, lncRNA *H19* harboring MREs for miR-138 and miR-200a positively regulates the expression of *VIM*, *ZEB1/2*, and *TWIST2*, which are known EMT regulators. Therefore, lncRNA *H19* potentiates metastasis by abolishing the function of EMT-suppressing miRNAs^31,32^.

Here, we identify *LINC00263*, also called oligodendrocyte maturation-associated long intergenic ncRNA (*OLMALINC*), as a novel hnRNPK-regulated lincRNA. And we also find that hnRNPK directly interacts with *LINC00263* and stabilizes *LINC00263*. ASO pull-down assays and small RNA sequencing revealed that miR-147a interacts strongly with *LINC00263*, indicating that *LINC00263* controls the repressive function of miR-147a. *LINC00263* is known to be upregulated in a wide range of cancer types including lung adenocarcinoma, colorectal cancer, and renal carcinoma^15^. Consistent with our observation, *LINC00263* was recently reported to be abnormally regulated in lung cancer^15^.

Comparison of RNA sequencing data and miR-147a target genes revealed that *CAPN2* is a downstream effector of hnRNPK/*LINC00263*/miR-147a. Mechanistically, *LINC00263* increases CAPN2 expression by functioning as a ceRNA of miR-147a, and thus potentiates malignant capabilities. CAPN2 is a calcium-dependent protease and known to play an important role in the proliferation and metastasis of cancer cells.^33,34^. It was also reported to function as an oncogene by inducing EMT and increasing expression of matrix metalloproteinase 9 (*MMP9*)^33,35^. Abnormal expression of CAPN2 is closely associated with poor prognosis of ovarian cancer patients^36^. Moreover, CAPN2 is also related to metastatic prostate cancer by potentiating proliferative and invasive capabilities^37,38^.

Our results demonstrate that *LINC00263* is a novel target of hnRNPK and functions as a ceRNA for miR-147a, a *CAPN2*-targeting miRNA. Therefore, increased expression of *LINC00263* in cancer cells suppresses the repressive effects of miR-147a, thereby increasing CAPN2 expression. CAPN2 controls the malignant properties of cancer cells partly through ERK and p70S6K. Taken together, the hnRNPK/*LINC00263*/miR-147a/CAPN2 axis identified in this study represents a promising target for the treatment of human cancer.

## Supplementary information

Supplementary figure 1

Supplementary figure 2

Supplementary figure 3

Supplementary figure 4

Supplementary figure 5

Supplementary figure 6

Supplementary figure 7

Supplementary figure 8

Supplementary figure 9

Supplementary figure 10

Supplementary figure 11-1

Supplementary figure 11-2

Supplementary tables

Supplementary figure legends

RNA sequencing data
