# Supplementary figures and images for: hnRNPK-regulated LINC00263 promotes malignant phenotypes through miR-147a/CAPN2
Source: Cell Death Dis. 2021 Mar 17;12(4):290. doi: 10.1038/s41419-021-03575-1 (PMC7969774; doi:10.1038/s41419-021-03575-1)

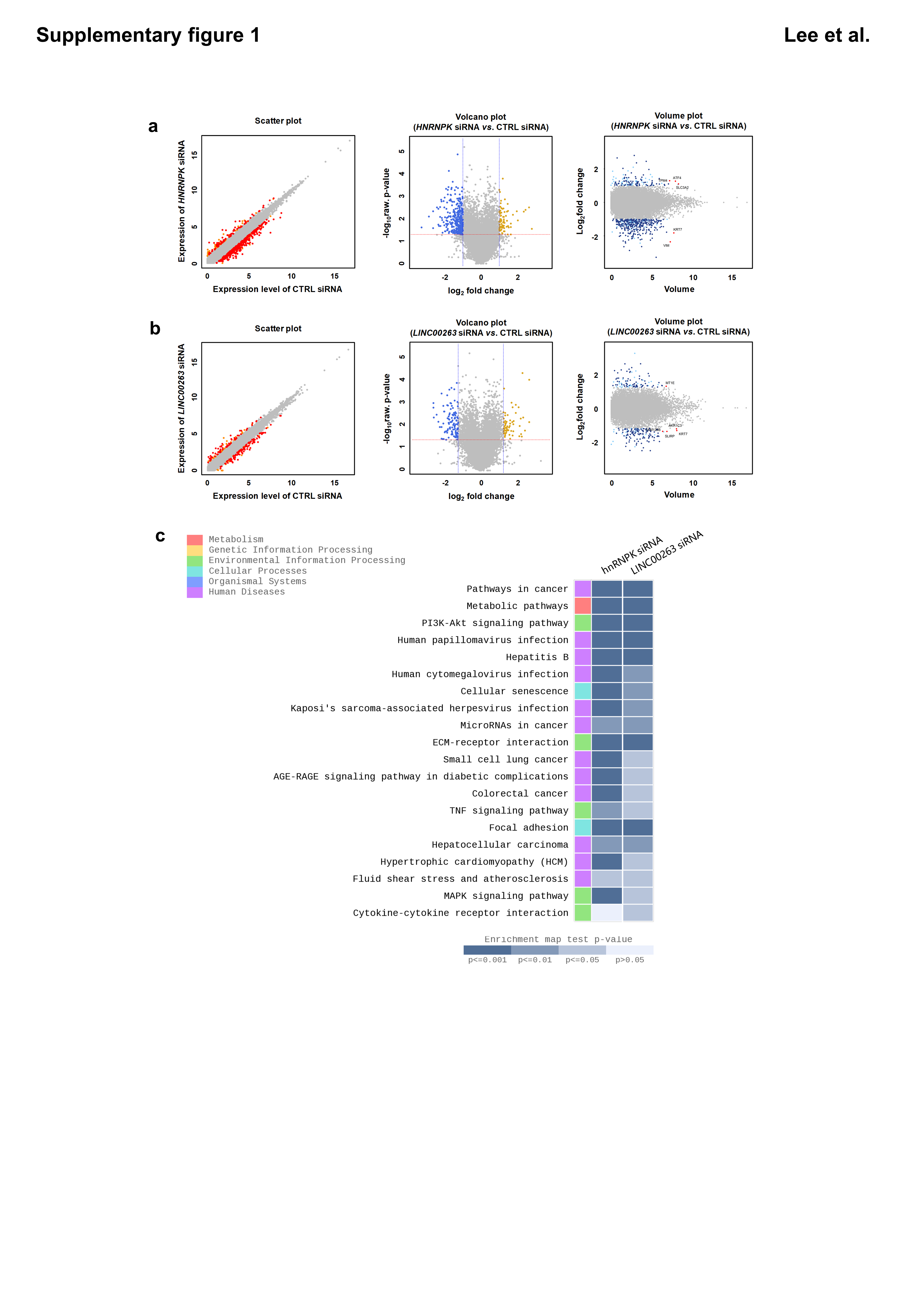

Supplement: Supplementary file 1 — Supplementary figure 1 [file 41419_2021_3575_MOESM1_ESM.tif]

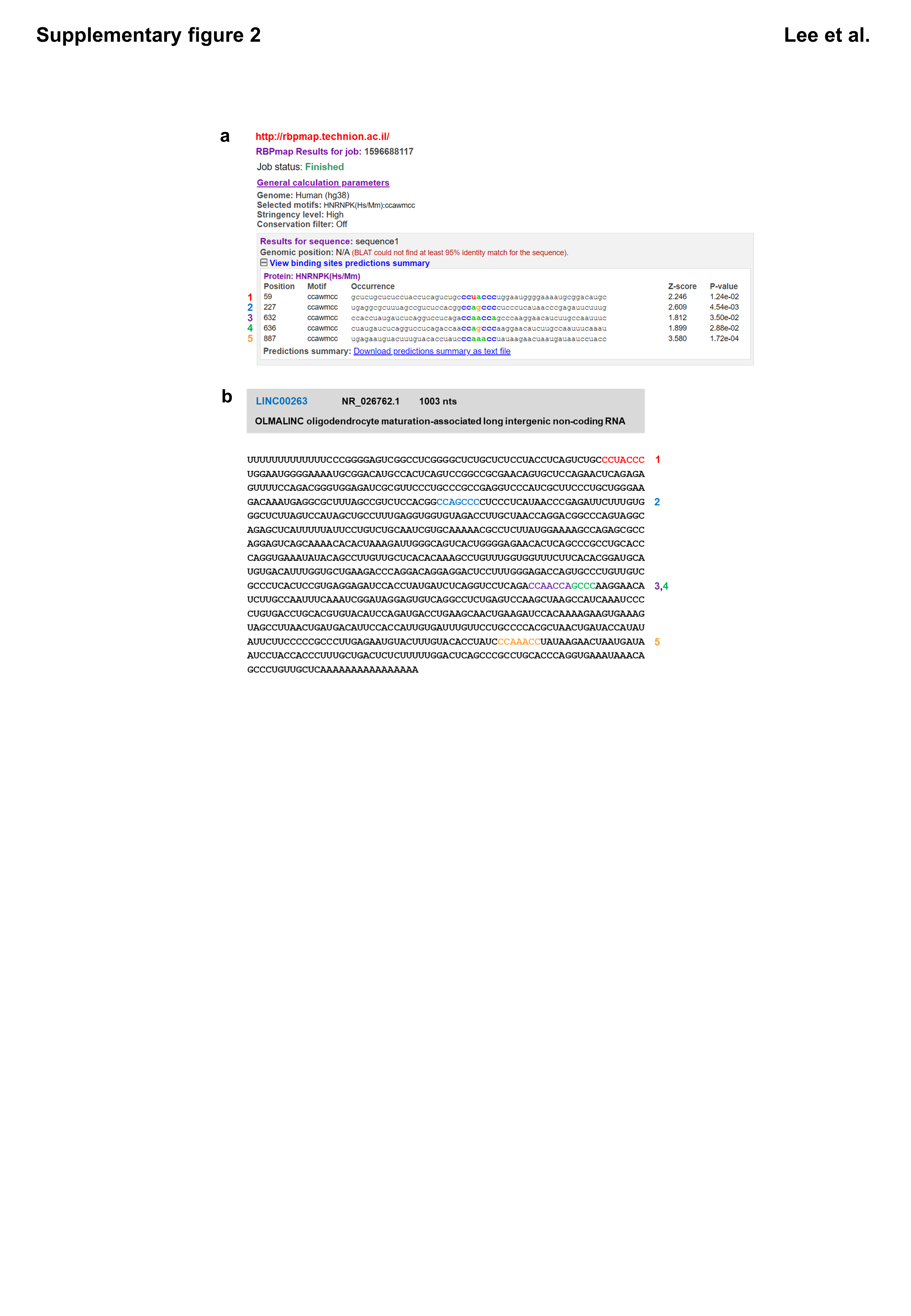

Supplement: Supplementary file 2 — Supplementary figure 2 [file 41419_2021_3575_MOESM2_ESM.tif]

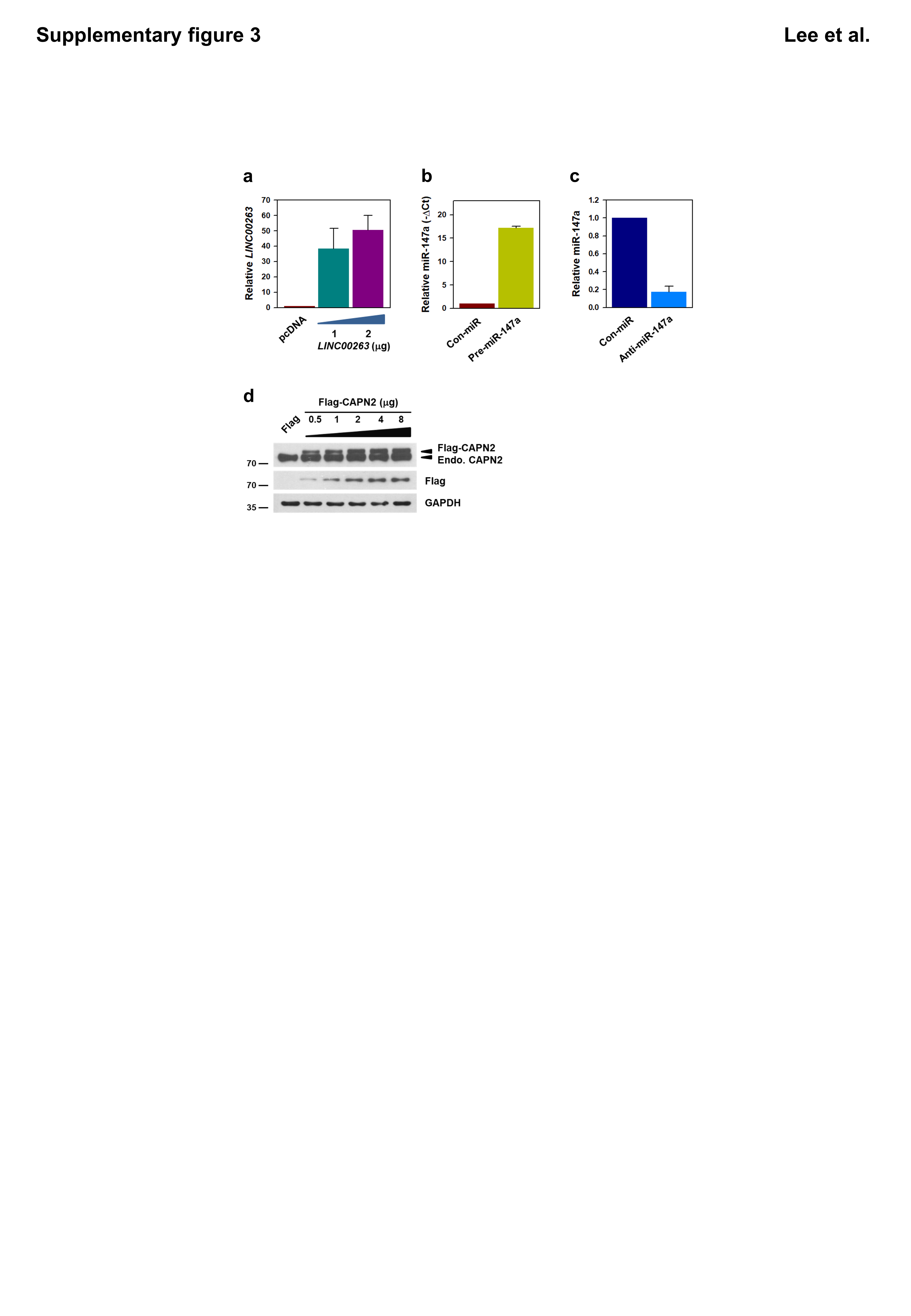

Supplement: Supplementary file 3 — Supplementary figure 3 [file 41419_2021_3575_MOESM3_ESM.tif]

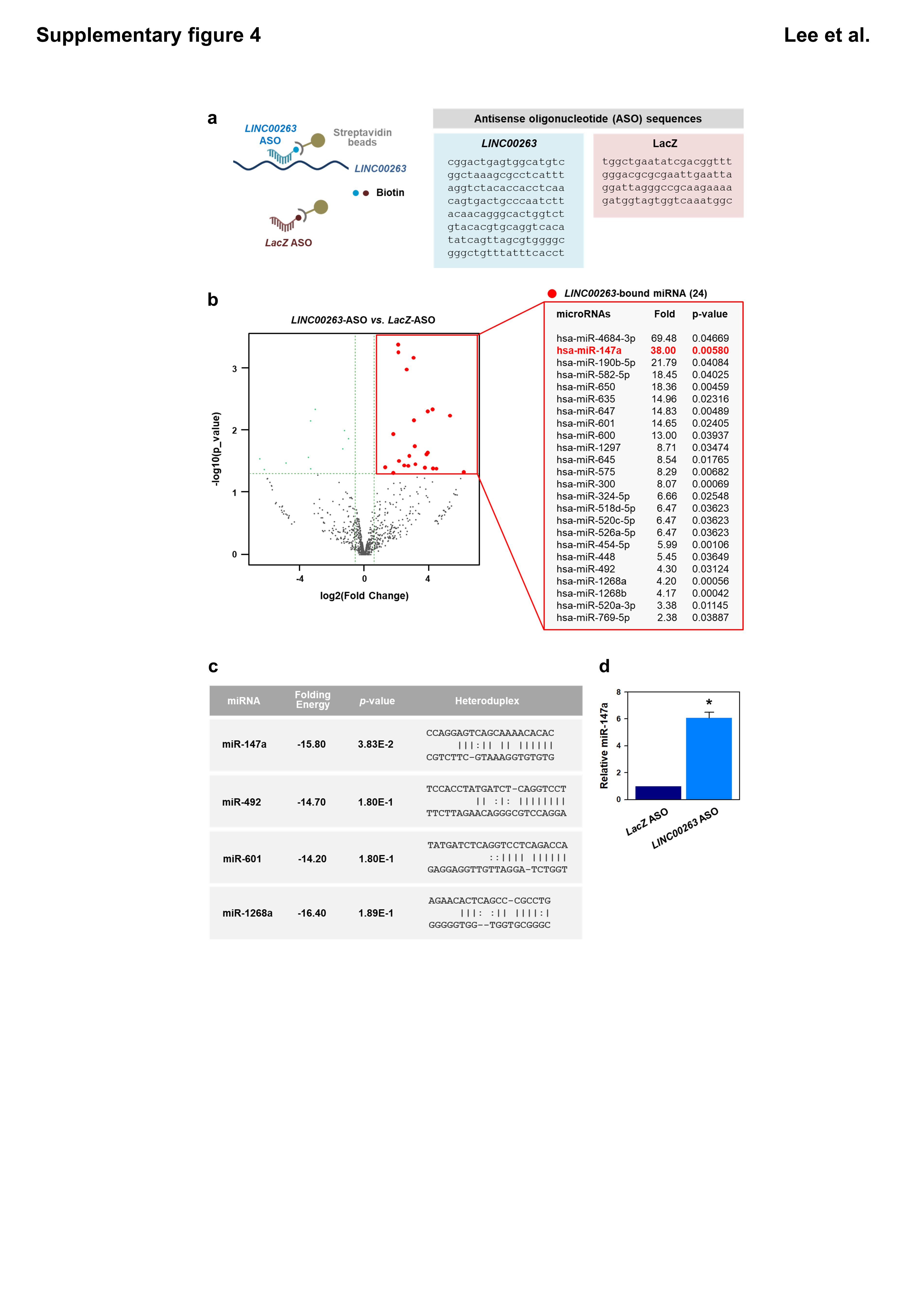

Supplement: Supplementary file 4 — Supplementary figure 4 [file 41419_2021_3575_MOESM4_ESM.tif]

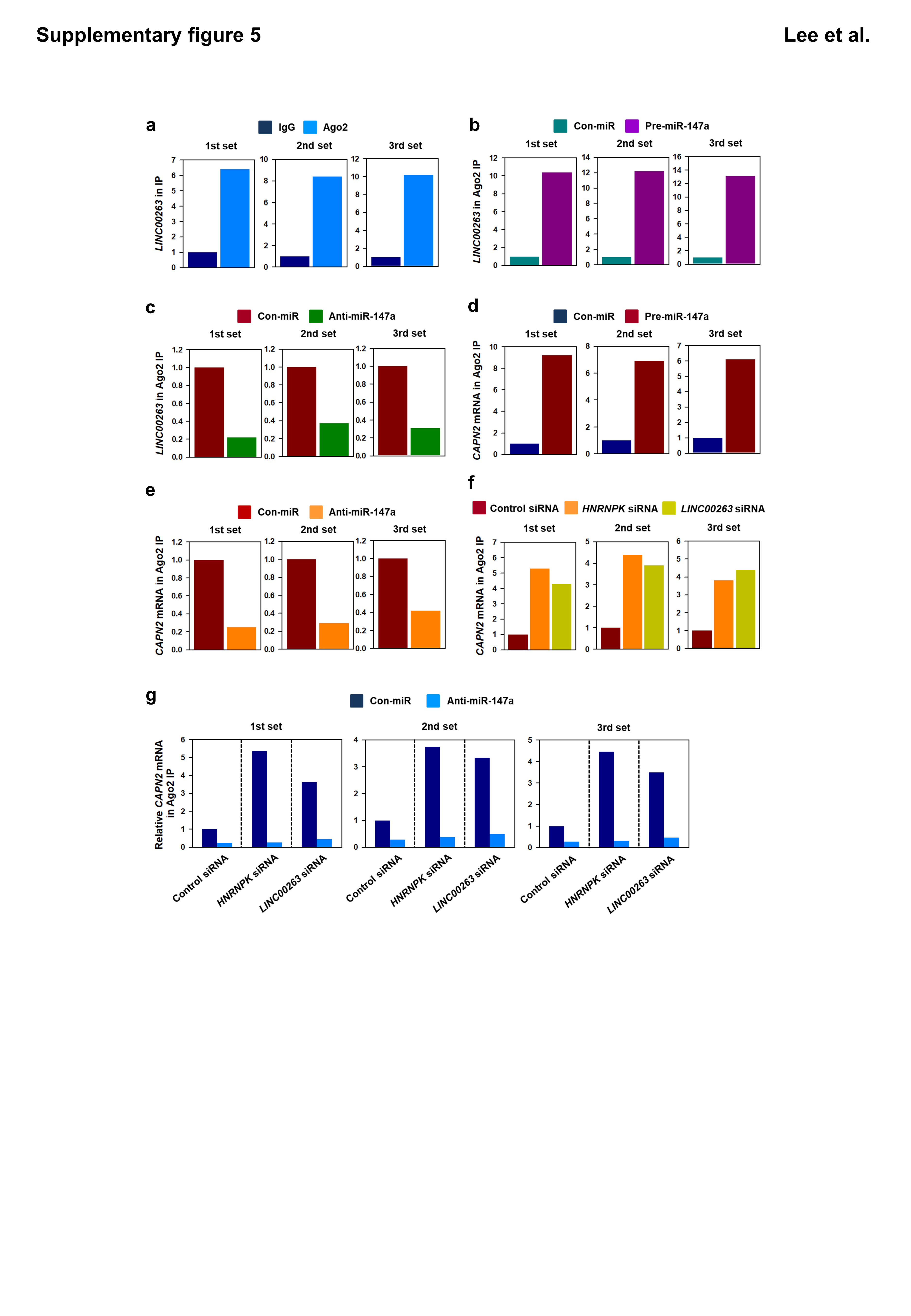

Supplement: Supplementary file 5 — Supplementary figure 5 [file 41419_2021_3575_MOESM5_ESM.tif]

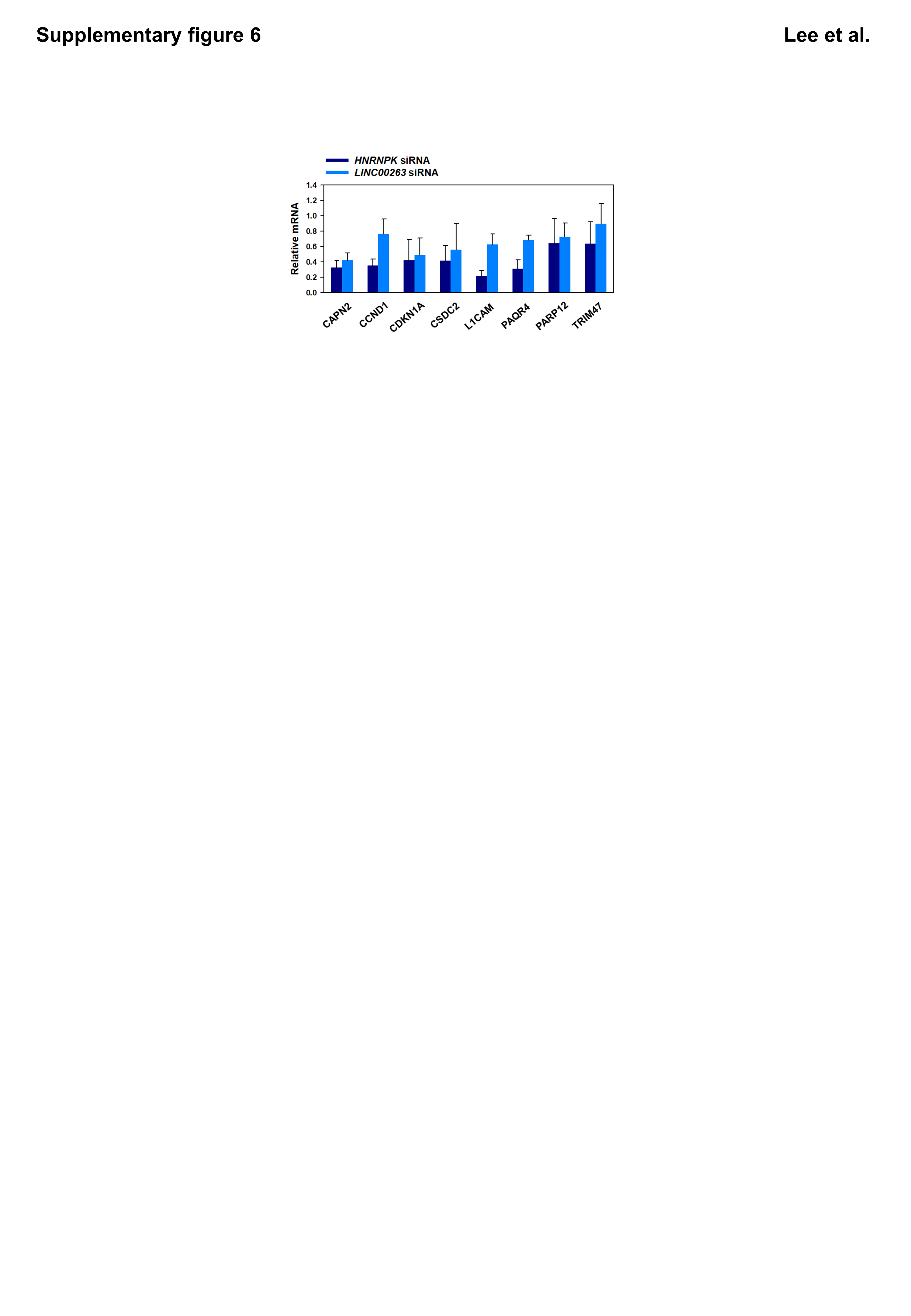

Supplement: Supplementary file 6 — Supplementary figure 6 [file 41419_2021_3575_MOESM6_ESM.tif]

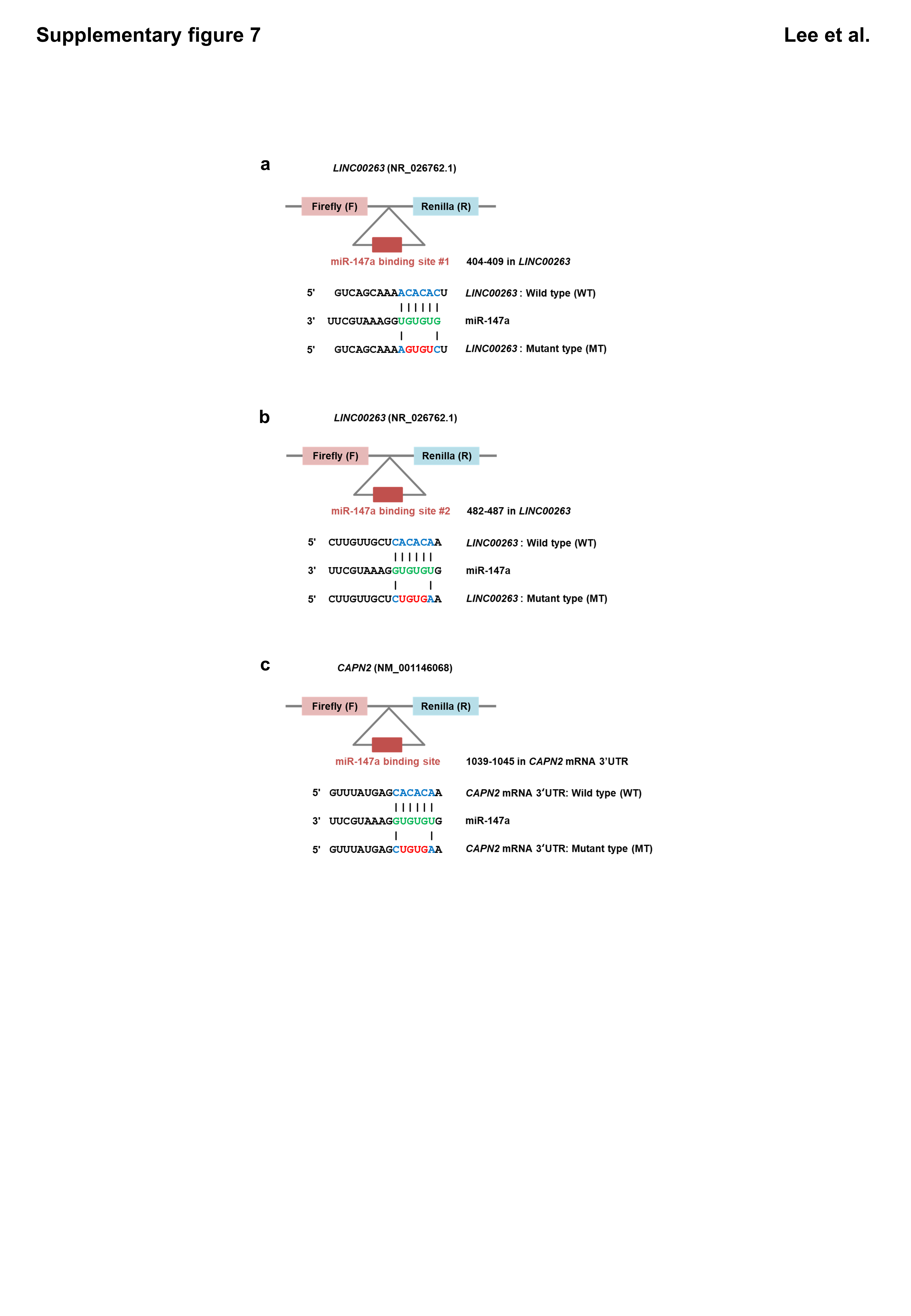

Supplement: Supplementary file 7 — Supplementary figure 7 [file 41419_2021_3575_MOESM7_ESM.tif]

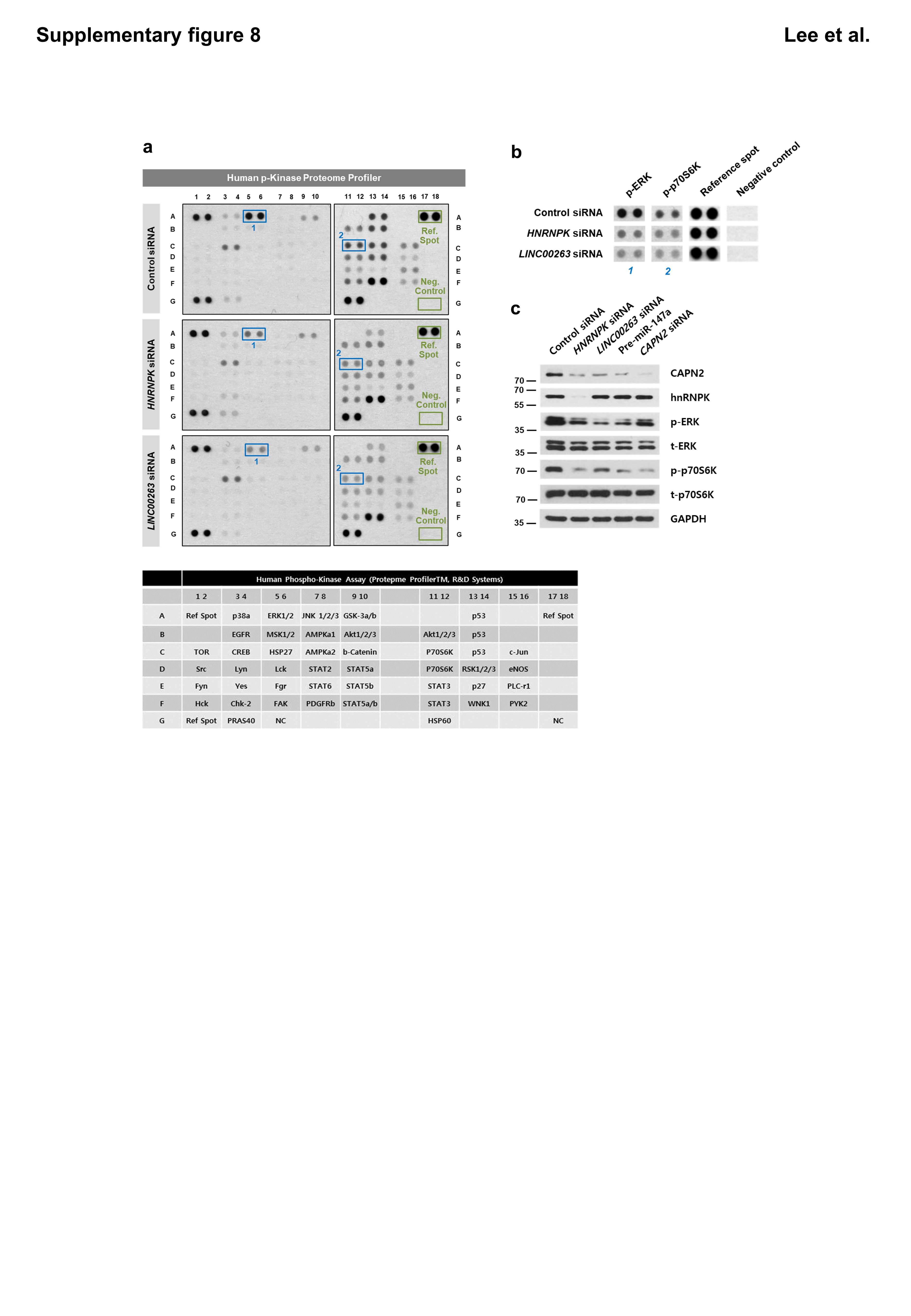

Supplement: Supplementary file 8 — Supplementary figure 8 [file 41419_2021_3575_MOESM8_ESM.tif]

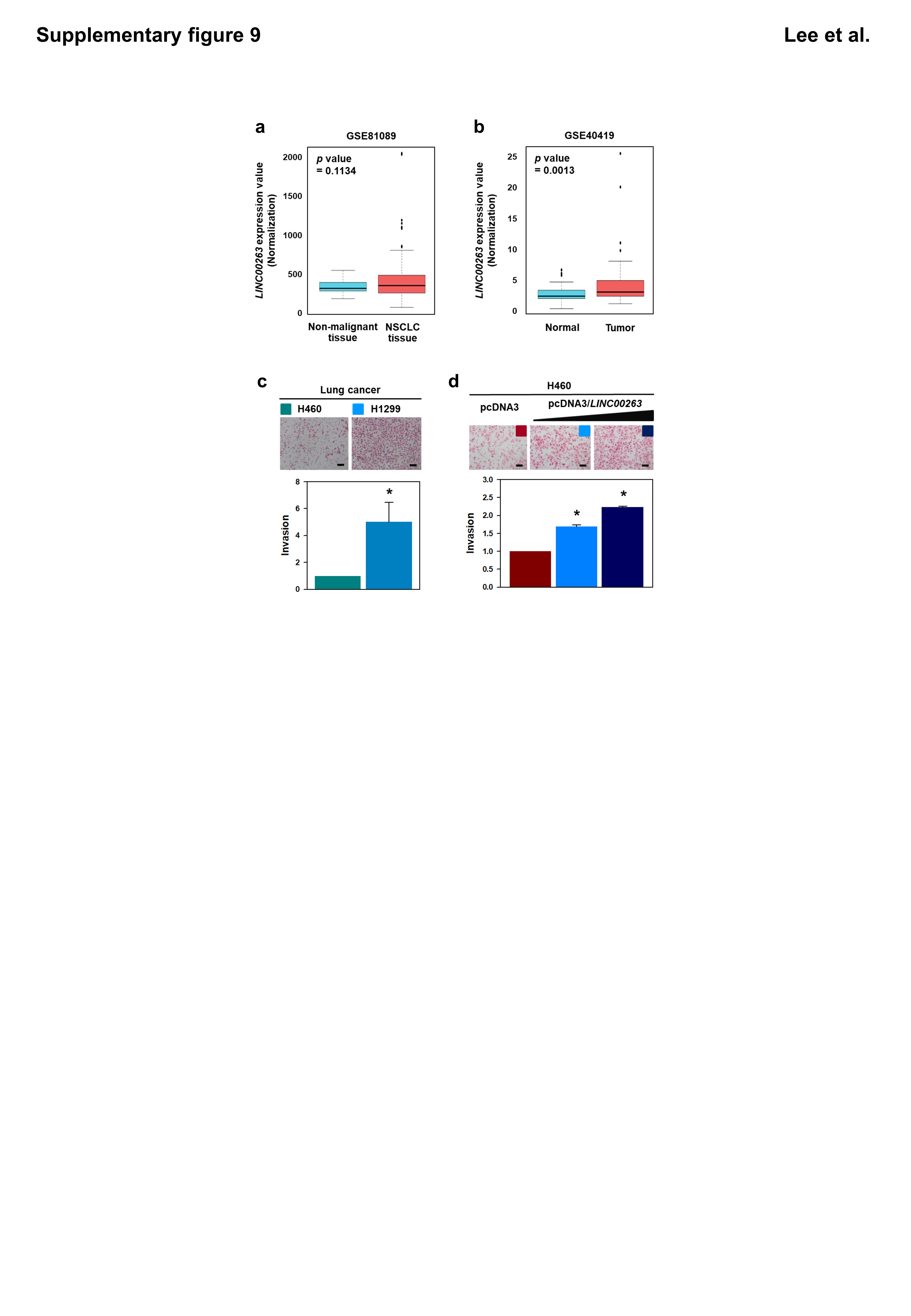

Supplement: Supplementary file 9 — Supplementary figure 9 [file 41419_2021_3575_MOESM9_ESM.tif]

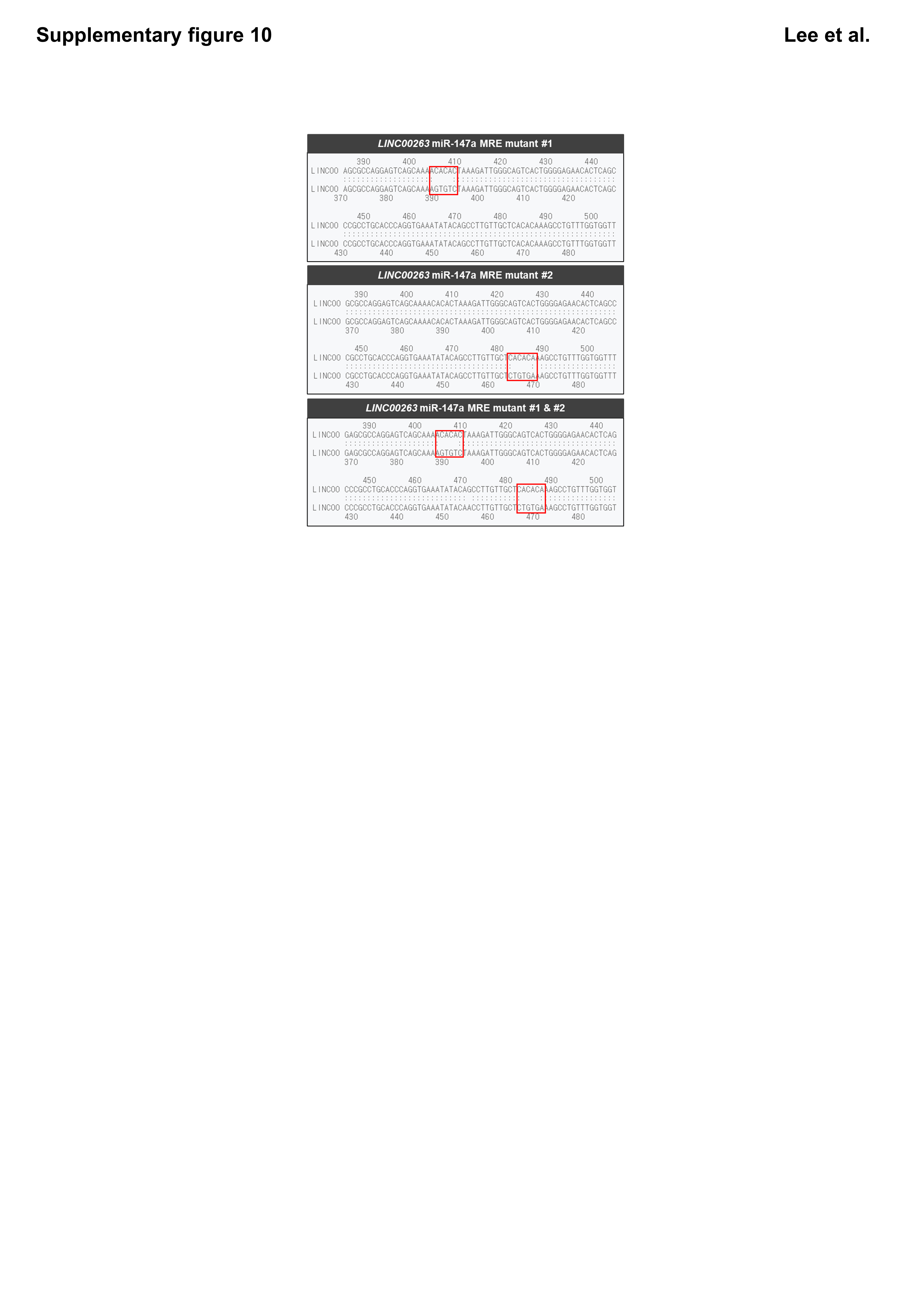

Supplement: Supplementary file 10 — Supplementary figure 10 [file 41419_2021_3575_MOESM10_ESM.tif]

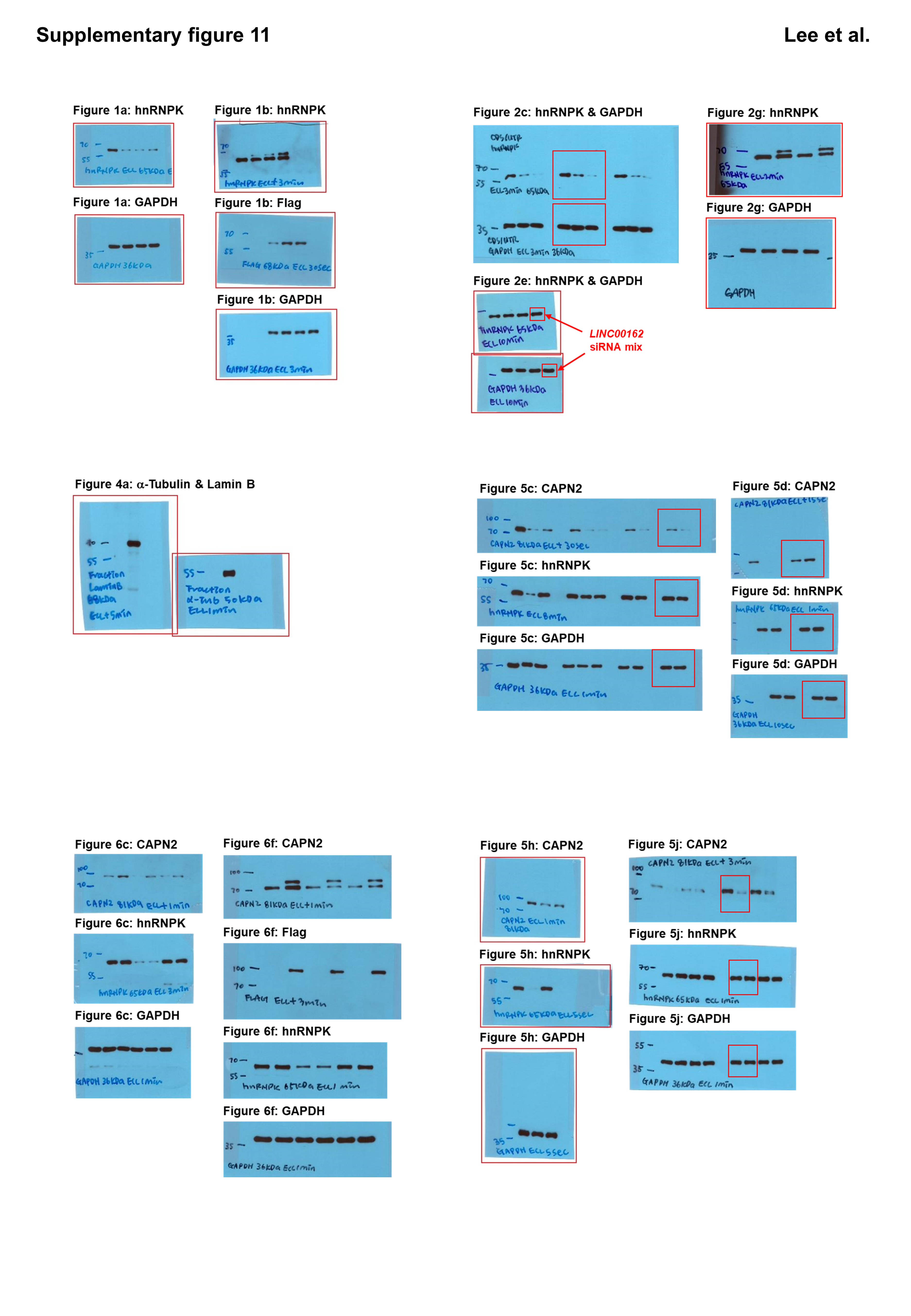

Supplement: Supplementary file 11 — Supplementary figure 11-1 [file 41419_2021_3575_MOESM11_ESM.tif]

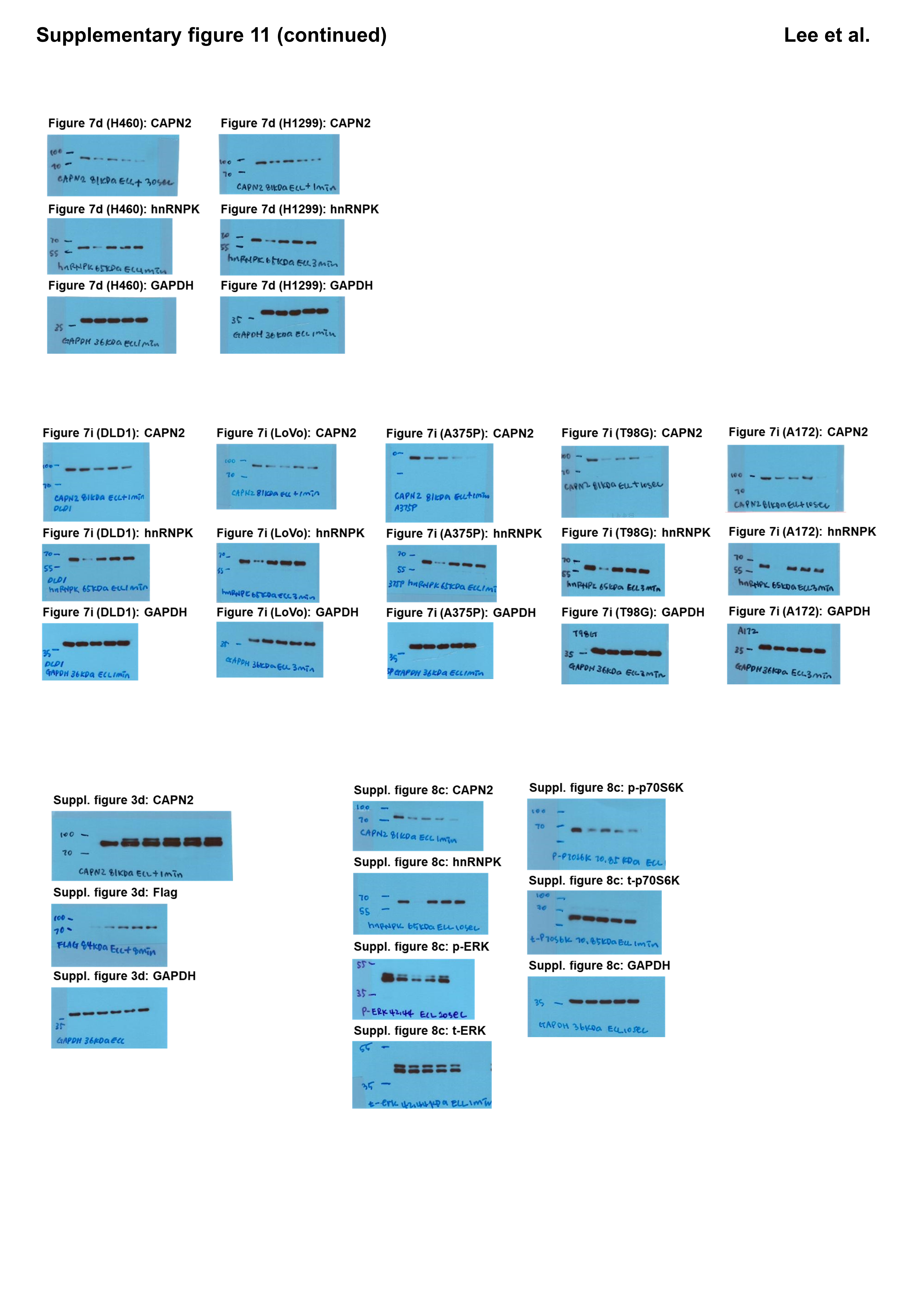

Supplement: Supplementary file 12 — Supplementary figure 11-2 [file 41419_2021_3575_MOESM12_ESM.tif]

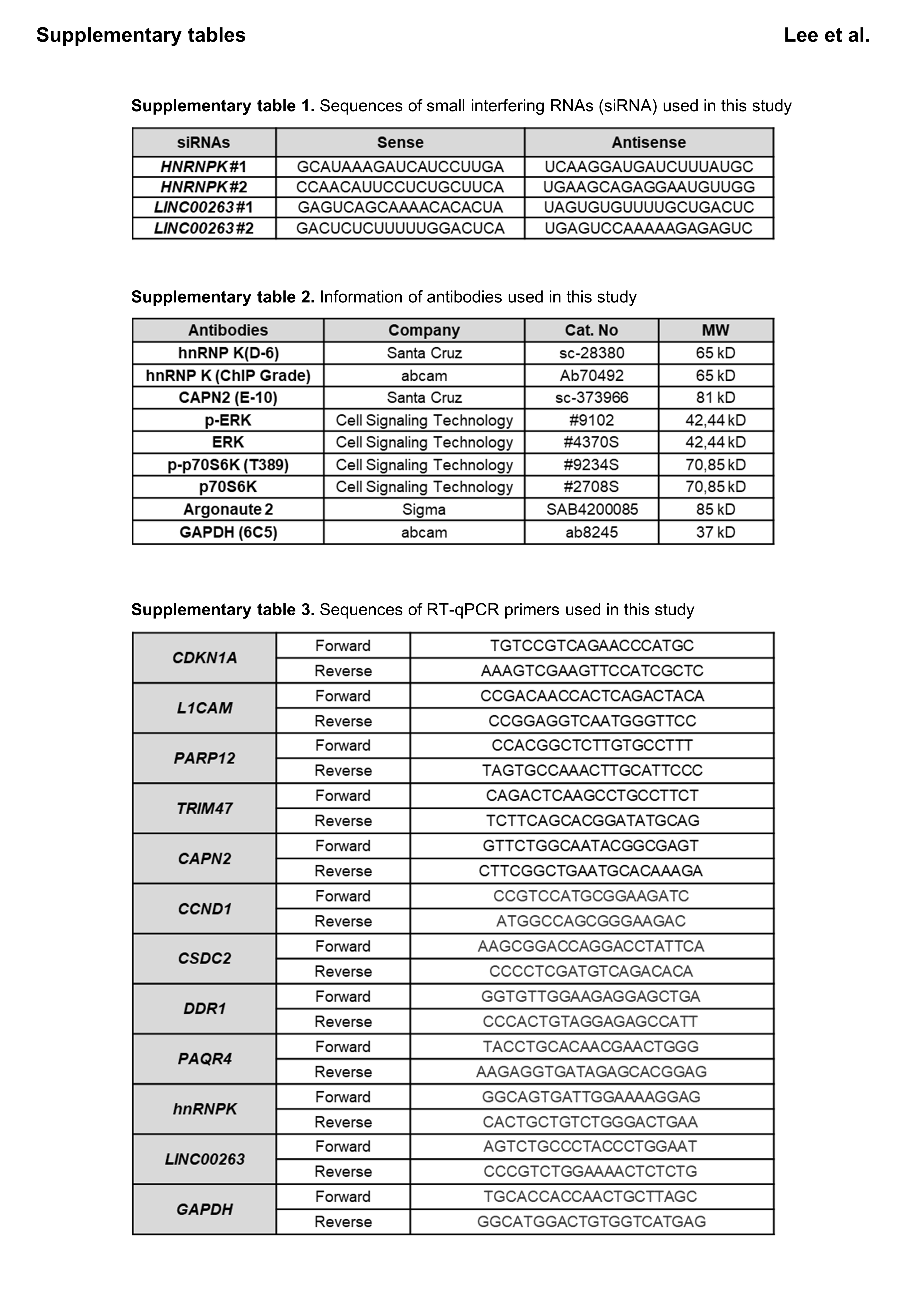

Supplement: Supplementary file 13 — Supplementary tables [file 41419_2021_3575_MOESM13_ESM.tif]
