# Supplementary material for: hnRNPK-regulated LINC00263 promotes malignant phenotypes through miR-147a/CAPN2
Source: Cell Death Dis. 2021 Mar 17;12(4):290. doi: 10.1038/s41419-021-03575-1 (PMC7969774; doi:10.1038/s41419-021-03575-1)
Supplement: Supplementary file 14 — Supplementary figure legends [file 41419_2021_3575_MOESM14_ESM.docx]

***Supplementary figure legends***

**Supplementary fig. 1 Analyses of RNA sequencing data.** (**a**-**b**) Various analyses plots, including scatter, volcano, and volume plots were generated corresponding to the RNA sequencing data obtained from hnRNPK- (**a**) and *LINC00263*-silenced HeLa cells (**b**). Gene ontology (GO) analyses are also shown (**c**).

**Supplementary fig. 2 Interaction of hnRNPK with *LINC00263* is dependent on KH1 and KH2 domains.** (**a**-**b**) Prediction of hnRNPK binding sites in LINC00263 using RBPmap (<http://rbpmap.technion.ac.il/>) was shown (**a**). Each binding locations and sequences were also presented (**b**).

**Supplementary fig. 3 Verification of overexpression and knockdown by RT-qPCR or Western blot analyses.** (**a**) Following the transfection of HeLa cells with overexpression vector (pcDNA/*LINC00263*), the expression of *LINC00263* was assessed by RT-qPCR analysis. (**b**-**c**) miR-147a overexpression by pre-miR-147a (**b**) and inhibition by anti-miR-147a (**c**) were verified by RT-qPCR analysis using miRNA-specific TaqMan primer. (**d**) To determine the efficiency of CAPN2 overexpression vector, HeLa cells were transfected with various concentrations of the vector and the level of CAPN2 was determined by Western blot analysis. For the rescue experiments, 4 μg of vector was used.

**Supplementary fig. 4 Identification of *LINC00263*-associated miRNAs by antisense oligonucleotide pull-down (ASO PD) followed by small RNA sequencing.** (**a**) Schematic of the ASO PD experiment and the sequences of *LacZ* and *LINC00263* ASOs used in this study. (**b**) Volcano plot of small RNA sequencing and 24 miRNAs enriched in *LINC00263* ASO PD that were selected as putative decoy targets of *LINC00263* are shown. (**c**) Using a bioinformatic tool (RNA22, <https://cm.jefferson.edu/rna22/Interactive>), four of the 24 miRNAs were predicted to strongly interact with *LINC00263*. Among them, miR-147a was selected for further studies based on its folding energy and significance. (**d**) To validate the interaction between *LINC00263* and miR-147a, we performed ASO PD experiments. Following biotin pull-down using *LacZ*-ASO or *LINC00263*-ASO, the level of miR-147a was determined by RT-qPCR analysis, using U6 levels for normalization.

**Supplementary fig. 5 Three individual results of RNP-IP experiments.** All RNP-IP results indicate the mean and standard deviation of the values obtained in three independent experiments. (**a**) *LINC00263* in Ago2 RNP-IP; (**b**) *LINC00263* in Ago2 RNP-IP with high expression of miR-147a; (**c**) *CAPN2* mRNA in Ago2 RNP-IP; (**d**-**e**) *CAPN2* mRNA in Ago2 RNP-IP with high (**d**) and low expression (**e**) of miR-147a; (**f**) *CAPN2* mRNA in Ago2 RNP-IP with knockdown of hnRNPK or *LINC00263*; (**g**) *CAPN2* mRNA in Ago2 RNP-IP in rescue experiments.

**Supplementary fig. 6 The levels of common target genes in hnRNPK- and *LINC00263*-silenced cells.** To test whether knockdown of hnRNPK and *LINC00263* decreased the expression of miR-147a target genes, the levels of common target mRNAs were examined by RT-qPCR analysis.

**Supplementary fig. 7 Schematic of the luciferase reporter vectors containing wild-type or mutant sequences of miR-147a MRE.** (**a**-**b**) Luciferase reporter vectors (wild-type and mutant) with miR-147a MREs in *LINC00263* sequence. Using bioinformatic tools, two MREs were identified in the *LINC00263* sequence: miR-147a binding site #1 and #2. (**c**) Luciferase reporter vectors (wild-type and mutant) with miR-147a MRE in the 3'UTR of *CAPN2* mRNA.

**Supplementary fig. 8 Search for intracellular signaling molecules linked to hnRNPK/*LINC00263*.** To identify the signaling molecules, the activation of which is involved in the oncogenic function of hnRNPK/*LINC00263*, the Proteome Profiler Human Phospho-Kinase Array Kit (R&D Systems, ARY003B) was used. Briefly, HeLa cells were transfected with *HNRNPK* or *LINC00263* siRNA and whole cell lysates were incubated with each array in which various capture antibodies were spotted. Phosphorylated signaling molecules were detected by incubating with biotinylated phospho-specific detection antibodies and then visualized using chemiluminescent reagents. (**a**) Total results of the Proteome Profiler Human Phospho-Kinase Array. (**b**) Selected spots that were decreased in the hnRNPK- and *LINC00263*-silenced cells: p-ERK and p-p70S6K. (**c**) HeLa cells were transfected with control siRNA, *HNRNPK* siRNA, *LINC00263* siRNA, pre-miR-147a, or *CAPN2* siRNA. Following preparation of whole cell lysates, Western blot analysis was performed using the indicated antibodies. The level of GAPDH protein served as a loading control.

**Supplementary fig. 9 Comparison of *LINC00263* expression level in lung cancer patients.** (**a**-**b**) The expression of *LINC00263* was compared between non-malignant and non-small cell lung cancer tissues (**a**, GSE81089), or normal and tumor tissues (**b**, GSE40419). (**c**) The invasive ability of two lung cancer cells was assessed by Transwell invasion assay. (**d**) Invasiveness of *LINC00263*-overexpressing H460 cells was determined by comparing the number of invading cells. Bars on microscopic images represent 100 μm. Statistical analyses were performed using the Student’s *t*-test using three independent experiments (* *p* < 0.05). All data represent mean ± standard variation (SD).

**Supplementary fig. 10 Construction of mutated *LINC00263* overexpression vectors.** Two miR-147a MREs are found in the sequence of *LINC00263*. As with constructing the luciferase reporter vectors, four nucleotides of each miR-147a MRE in *LINC00263* were changed to block the binding of miR-147a.

**Supplementary fig. 11 Uncropped Western blot images shown in this manuscript.**

***Supplementary tables***

**Supplementary table 1 Sequences of small interfering RNAs (siRNAs) used in this study.**

**Supplementary table 2 Information about the antibodies used in this study.**

**Supplementary table 3 Sequences of the RT-qPCR primers used in this study.**
